# Genome-Wide Transcriptional Profiling and Metabolic Analysis Uncover Multiple Molecular Responses of the Grass Species *Lolium perenne* Under Low-Intensity Xenobiotic Stress

**DOI:** 10.3389/fpls.2015.01124

**Published:** 2015-12-17

**Authors:** Anne-Antonella Serra, Ivan Couée, David Heijnen, Sophie Michon-Coudouel, Cécile Sulmon, Gwenola Gouesbet

**Affiliations:** ^1^Centre National de la Recherche Scientifique, Université de Rennes 1, UMR 6553 ECOBIORennes, France; ^2^Centre National de la Recherche Scientifique, Université de Rennes 1, UMS 3343 OSURRennes, France

**Keywords:** RNA-Seq, glyphosate, tebuconazole, no observable adverse effect, residual pollution, perennial ryegrass, SnRKs

## Abstract

*Lolium perenne*, which is a major component of pastures, lawns, and grass strips, can be exposed to xenobiotic stresses due to diffuse and residual contaminations of soil. *L. perenne* was recently shown to undergo metabolic adjustments in response to sub-toxic levels of xenobiotics. To gain insight in such chemical stress responses, a *de novo* transcriptome analysis was carried out on leaves from plants subjected at the root level to low levels of xenobiotics, glyphosate, tebuconazole, and a combination of the two, leading to no adverse physiological effect. Chemical treatments influenced significantly the relative proportions of functional categories and of transcripts related to carbohydrate processes, to signaling, to protein-kinase cascades, such as Serine/Threonine-protein kinases, to transcriptional regulations, to responses to abiotic or biotic stimuli and to responses to phytohormones. Transcriptomics-based expressions of genes encoding different types of SNF1 (sucrose non-fermenting 1)-related kinases involved in sugar and stress signaling or encoding key metabolic enzymes were in line with specific qRT-PCR analysis or with the important metabolic and regulatory changes revealed by metabolomic analysis. The effects of pesticide treatments on metabolites and gene expression strongly suggest that pesticides at low levels, as single molecule or as mixture, affect cell signaling and functioning even in the absence of major physiological impact. This global analysis of *L. perenne* therefore highlighted the interactions between molecular regulation of responses to xenobiotics, and also carbohydrate dynamics, energy dysfunction, phytohormones and calcium signaling.

## Introduction

Modern agriculture uses large amounts of phytosanitary products to maximize crop production. These products are mainly pesticides or mixtures of pesticides, which have been developed to repel, attenuate or kill pests and competitive plants (Arias-Estévez et al., 2008). However, runoff, leaching, or spray drift lead to the entry of a large fraction of pesticides into environmental compartments (soil, water, sediment, or atmosphere). These diffuse and residual contaminations are composed of mixtures of parent compounds, of their degradation products, and also of associated adjuvants (Helander et al., 2012) and induce many environmental impacts (Patty et al., 1997; Köhler and Triebskorn, 2013). Pesticides and related-degradation products chemically stress many non-target organisms in natural ecosystems, among which plants are particularly affected as sessile organisms (Child et al., 1993; Serra et al., 2013, 2015).

Mechanisms and regulations of chemical stress responses may differ between species as a result of micro- and macroevolutionary processes (Medina et al., 2007) and may influence the sensitivity or tolerance of plants and thereby their capabilities to maintain growth and development in polluted areas. Moreover, herbicide efficiency is increasingly affected by the emergence of resistance processes (Délye, 2013). Target-site resistance (TSR) results from gene mutations that alter herbicide targets, whereas processes of non-target-site resistance (NTSR) can result from multiple mechanisms of detoxification, tolerance or regulation (Délye, 2013). In the last decades, large-scale pesticide use on agricultural lands, resulting in long-term contamination (Singh et al., 2004), has put strong ecological and evolutionary pressure on the dynamics of plant communities, leading to resistance emergence (Délye, 2013). Widespread agricultural weeds are controlled by herbicide applications, which result in recurrent exposition for the surrounding vegetation generally composed of a majority of grasses. Among these species, several populations of *Lolium perenne* have been described as resistant to herbicides. Recently, it has been demonstrated that, in a population that displays glyphosate resistance, other mechanisms than mutation in the target site of glyphosate, the plastidic enzyme 5-enolpyruvylshikimate-3-phosphate synthase (EPSPS), were involved (Salas et al., 2015). Similarly, resistance to the acetolactate-synthase (ALS) inhibiting herbicide pyroxsulam in *Lolium* sp. populations involves a NTSR response implying differential gene expression and different mechanisms that remain to be elucidated (Duhoux et al., 2015). Multiple-herbicide resistance has also been described in a specific population of *Lolium perenne* spp. *multiflorum*, but although some resistances to chemical groups of ALS inhibitors and triazine have been explained by the presence of mutations in target genes, none is responsible for the resistance of this population to glyphosate (Liu et al., 2013a), showing the complexity of NTSR and underlying mechanisms.

The mechanisms of plant responses to chemical stress induced by xenobiotics are not fully understood. Such mechanisms are often studied under conditions of high exposures corresponding to application levels in the field. High levels of xenobiotics strongly impact organism physiology and defense responses, by inducing molecular injury and damages (Teixeira et al., 2007; Ramel et al., 2009; Das et al., 2010; Nobels et al., 2011; Gomes et al., 2014), mainly related to oxidative stress, membrane disruption, lipid peroxidation, protein damage, or DNA damage. In contrast, few studies deal with the responses to conditions of low exposure to pesticides in a context of runoff or residual contaminations. It has been shown that long-term exposure to sub-lethal pesticide level impacts plant community at the plant development level without causing mortality (Pfleeger et al., 2012). Londo et al. (2014) demonstrated that biomass, flowering phenology, and reproductive functions are affected by sub-lethal glyphosate exposure in *Brassica* spp. Moreover, Ivanov et al. (2013) observed that although sub-lethal concentrations of atrazine did not cause immediate negative and visible effect, long-term exposition impacted the redox homeostasis through an oxidative stress. Long and low herbicide exposure results also in rapid herbicide resistance evolution for exposed populations as demonstrated by Yu et al. (2013) for *Lolium rigidum* in presence of diclofop-methyl. At the molecular level, Das et al. (2010) demonstrated by genome-wide expression profiling that five commercial herbicide formulations at concentration producing a 50% reduction in shoot dry weight (EC50, sub-lethal levels) specifically affected the expression of genes related to ribosome biogenesis and translation, secondary metabolism, cell wall modification and growth. A very recent study demonstrated that subtoxic levels of herbicides acted as chemical hybridization agents, leading to male sterility for the production of hybrid seeds. Their effects were related to reprogramming of gene expression and metabolism in response to low-level herbicide treatments (Li et al., 2015). This study thus showed that complex mechanisms of low-intensity herbicide stress responses may exist. ^1^H NMR fingerprinting was also undertaken to analyse substantial metabolic changes in *Lemna minor*'s metabolome after short exposure to different pesticide treatments leading to no phytotoxicity symptom in attempt to develop novel ecotoxicological biomarkers. The discrimination between treatments was mostly based on metabolic variations of substances containing methylene, methine, hydroxy, amine, thiol, olefin, and aldimine groups without mechanistic conclusions (Aliferis et al., 2009). Complex mixtures of xenobiotics at realistic environmental levels produced different effects than those caused by compounds alone or by simple addition of effects (Lydy et al., 2004). Depending on the chemical properties and modes of toxic action of each compound, mixtures can result in higher (synergism) or weaker (antagonism) toxicity, as highlighted for various organisms, as humans, invertebrates or plants (Hertzberg and MacDonell, 2002; Hernández et al., 2012; Cedergreen, 2014). Frankart et al. (2002) have thus shown synergistic effect of a mixture of herbicide (flumioxazin) and copper on photosynthesis inhibition in *L. minor* whereas a mixture of fungicides (fludioxonil or procymidone) and copper produced an antagonism effect. Mixture effects are difficult to analyse and to predict (Dévier et al., 2011; Serra et al., 2013, 2015), and interactions between compounds can alter bioavailability or uptake rate and transport, metabolic activities, target site binding and/or compound excretion (Cedergreen, 2014). Their study remains however of interest, in particular in the case of no observed effect individual concentrations (Walter et al., 2002).

Hormetic effects and safener effects indicate that xenobiotics can also affect plants under conditions of no adverse effect (NOAE situation: No Observable Adverse Effect) through mechanisms that have seldom been investigated. Hormetic effects that induce beneficial impacts by exposure to low doses of a potentially toxic stressor are achieved through the activation of signal and regulation pathways independently of cellular damage (Velini et al., 2008; Costantini et al., 2010; Belz and Duke, 2014). In that context Nadar et al. (1975) described in Sorghum the growth-promoting effect of atrazine at sub-lethal concentrations in relation with cytokinin-like activity. Stamm et al. (2014) demonstrated in soybean that, even though a thiamethoxam seed treatment did not significantly impacted shoot height and plant biomass, the expression of genes related to plant defense and stress response was altered. Thus, the use of Cruiser® 5FS induces unexpected effects, regarded as cryptic, on a non-target organism. Such cryptic effects were observed in *Arabidopsis thaliana* by Serra et al. (2013) who analyzed the effects of low doses of pesticides, of pesticide degradation products and of their mixtures. In this study, AMPA and hydroxyatrazine, the main degradation products of glyphosate and atrazine, respectively, led to NOAE situations, and nevertheless had significant effects on the expression of genes already known to be affected by high pesticide exposure and on metabolic profiles (Serra et al., 2013). Some chemical treatments induced extensive metabolic changes, such as accumulation of stress-related metabolites (ascorbate) and decrease of carbohydrate levels. Moreover, these chemical stresses effects occurred in parallel with modifications of hormone-related and transcription regulation-related gene expression, thus suggesting underlying regulatory actions of xenobiotic compounds (Serra et al., 2013). Strong interaction effects between chemicals at the molecular, metabolic and physiological levels confirmed that pesticide-related products may act on regulation pathways (Serra et al., 2013).

An another integrative study, focusing on the physiological and metabolic responses of *L. perenne* to diverse subtoxic conditions of chemical stresses, showed primary effects of chemical stressors on seedling metabolism, physiology and growth (Serra et al., 2015). A short exposure to low doses of glyphosate, tebuconazole and their mixture, which consisted of transfer exposure on chemical stressors containing medium during 4 days, did not have any negative effect on root length (NOAE situation), root growth being the most sensitive physiological parameter, but caused cryptic effects on metabolic, regulatory, and signaling processes (Serra et al., 2015). These effects did not however translate into long-term loss of fitness, thus indicating a situation of tolerance to low-level chemical stress. Short exposure was associated with unexpected metabolic changes, as for example significant decrease of Suc and Glc, corresponding to major reorientation of central carbon metabolism. The global analysis in *L. perenne*, combining leaves and root metabolites after short exposure and direct and long exposure, allowed to demonstrate that responses to low chemical stress were associated through a complex network of metabolic correlations converging on Asn, Leu, Ser, and glucose-6-phosphate (Glc-6-P), which could potentially be modulated by differential dynamics and interconversion of soluble sugars (Suc, trehalose, and Glc; Serra et al., 2015). Such complex metabolic changes reflected chemical stress adjustment rather than deregulation of homeostasis, then leading to root growth maintenance, even under long-term exposure, and thus suggesting the implication of primary mechanisms and molecular regulations (Serra et al., 2015). More importantly, these analyses suggested that complex signaling networks may directly participate in chemical stress responses to rapidly adjust plant metabolism and to counteract mild damaging stress. Moreover, the discovery of major cryptic effects on metabolic, regulatory, and signaling mechanisms under such NOAE conditions raises the issue of the stress concept in plants, as outlined by other authors (Kranner et al., 2010). Within this context, the varying levels of plant stress responses to varying stress intensity may be adaptive. In the absence of harmful effects, a “mild” stress can induce an “alarm response” characterized by post-translational and stress signaling resulting in transcriptomic modifications. The characterization of such low-intensity adaptive mechanisms is therefore of utmost importance, with a number of potential agronomical and ecological applications (stress shield concept).

*L. perenne* is a perennial species of high ecological and agronomic interest. It is one of the predominant forage grasses of high quality in temperate areas (Casler and Duncan, 2003; Barbehenn et al., 2004). Carbon sequestration, soil formation and nutrient cycling are improved by *L. perenne* cover crops (Pouyat et al., 2009). Its value also resides in its relative tolerance to various pollutants of different chemical nature (Dear et al., 2006), which explains why it has been used for phytoremediation strategies (Bidar et al., 2009; D'Orazio et al., 2013).

The present work analyses the molecular responses of *L. perenne*, using a transcriptomic approach, in order to characterize the mechanisms underlying responses to mild chemical stresses induced by no-adverse-effect doses of pesticides. In order to decipher primary mechanisms and pathways involved in adjustments, stress treatments consisted in transfer experiments of non-stressed plants to xenobiotic-containing medium during short exposure. Pesticide treatments consisting of glyphosate, tebuconazole and their combination were applied at NOAE levels to the roots of *L. perenne* seedlings. Both pesticides have been found at residual levels in soils of field margins (Serra et al., 2013) and are frequently detected in runoff water (Potter et al., 2014; Sasal et al., 2015). They are representative examples of agricultural pollution. The impact of root exposure on the whole plant is therefore a primordial process during plant/xenobiotic interactions under conditions of edaphic xenobiotic pollution. Glyphosate is a broad spectrum herbicide (Duke and Powles, 2008) which disrupts the synthesis of aromatic amino acids by inhibiting EPSPS, a key enzyme in the shikimate pathway (Steinrücken and Amrhein, 1980). Tebuconazole, besides acting and being used as a fungicide, can inhibit sterol 14α-demethylase enzymes (Lamb et al., 2001) and limit the rate of gibberellin biosynthesis (Child et al., 1993) in plants. Transcriptomic analysis of *L. perenne* responses to chemical stress was carried out by RNA-Seq approach involving the pyrosequencing of leaf cDNA libraries (Huang et al., 2012; Ward et al., 2012). A *de novo* transcriptome of *L. perenne* was obtained by the assembly of cDNA reads. A *de novo* assembly was performed using model species data as there is no reference genome available for *L. perenne*. Differential expression of genes and functions were analyzed in parallel with metabolic data in order to obtain a functional insight of molecular regulations induced by low-intensity chemical stress. Our present study shows that, taken together, the effects of pesticide treatments on metabolites and gene expression strongly suggest that low levels of pesticides, whether as single molecule treatment or as mixture, interact, even in the absence of major physiological impact, with plant cell functions as carbohydrate regulations and signaling. The characterization of plant/xenobiotic direct interactions with signaling and hormone cross-talk effects, which is a major field of research in animal toxicology (Frye et al., 2011), should provide novel insights into the environmental impact of low-level runoff or persistent pesticides on plant communities.

## Materials and methods

### Plant material and growth conditions

Seeds of *L. perenne* (Brio cultivar) were briefly washed in ethanol and surface-sterilized in bayrochlore (20 g L^−1^ in water) containing 0.05% tween (v/v) for 20 min and rinsed five times in sterilized water. Moistened seeds were placed in Petri dishes in the dark at 4°C for 7 days in order to break dormancy and homogenize germination. Seeds were sown on pieces of gauze and placed at the top of sterile culture tubes containing liquid growth medium. Gauze pieces were continuously moistened by soaking gauze edges into culture medium, in order to maintain humidity for germination. Germination and growth were carried out under axenic conditions in a control growth chamber at 22°C/20°C under a 16 h light (6000 lux)/8 h dark regime. Growth medium consisted of Hoagland basal salt mix (N°2, Caisson Laboratories, North Logan, UT, USA) adjusted to pH 6. Transfer experiments consisted in xenobiotic exposure of young plants at the same stage of photosynthetic development. After 7 days of growth under control conditions, seedlings were transferred to fresh growth medium containing chemical stressors, thus resulting in xenobiotic exposure at root level. Shoots were harvested 4 days later, corresponding to 11 days of total growth. Different chemical treatments were applied: the broad-spectrum herbicide glyphosate (G, 1 μM), the fungicide tebuconazole (T, 4 μM) and a combination of glyphosate and tebuconazole (GT, 1 μM and 4 μM, respectively). Leaves of seedlings were collected just before the start of the daylight period, ground in liquid nitrogen and stored at −80°C until use.

### Transcriptome sequencing

Five independent biological replicates, consisting in aerial parts (50 mg fresh weight) of 10 plantlets each, were harvested after transfer experiment and used for transcriptome sequencing. RNA was extracted using TRI Reagent® (Sigma) with an additional DNase treatment. Total RNA samples from each replicate were pooled per treatment condition [Control (C), glyphosate (G), tebuconazole (T) and glyphosate and tebuconazole mixture (GT)] and resulting samples were polyA-enriched with the Oligotex mRNA kit (Qiagen Cat. N°. 70042). Then, 250 ng of mRNA-enriched RNA were fragmented (ZnCl_2_), and reverse-transcribed to cDNA using cDNA Synthesis System kit (Roche Cat. N°. 11117831001). For each treatment, one Roche 454 library was prepared and sequenced twice on a quarter of plate on a Roche 454 GS-FLX, using titanium chemistry (titanium chemistry, Sequencing Kit XL+) at the Biogenouest core facility (Rennes, France). The sequencing data are available in the NCBI Sequence Read Archive (SRA) database under the accession reference PRJNA287779 (http://www.ncbi.nlm.nih.gov/Traces/sra/sra.cgi).

### Assembly and read processing

Read filtering and final assembly were performed using the Galaxy instance (Goecks et al., 2010). The 454 reads were processed to remove sequences below 250-bp and above 1000-bp. Reads were improved according to quality and by removing adapter sequences, undetermined bases (“N”) and poly-A/T tails. For generation of the reference transcriptome, selected clean reads from all libraries were pooled and assembled to improve mRNA lengths and produce a global library. Reads were assembled in contigs using the *de novo* assembly program Trinity (Haas et al., 2013). Trinity is an Illumina/Solexa-specialized transcriptomic assembler, which is suitable for non-strand-specific and single-end-read data. According to Ren et al. (2012), it gives the best performance among the multiple de Bruijn graph assemblers, and represents an alternative solution for reconstructing full-length transcripts from 454 reads. The assembly was conducted using the default parameters. Reads were ascribed to contigs using RSEM (RNA-seq by expectation-maximization) software and filtered according to the reads per kilobase of target transcript length per million reads mapped (RPKM) values equal to 1 or greater. After a first Trinity assembly, the unmapped (172,634 total unmapped sequences), and unused reads, were submitted again to the Trinity program, leading to a new assembly. Reads that again did not fit into contigs (42,351 unmapped reads) were defined as singletons. These unique sequences were added to the 14,811 unique contigs to constitute the reference transcriptome. The resulting singletons and contigs represented the candidate unigene set.

### Annotation and functional analyses

After assembling, tBLASTx alignments (*e* < 10e-5) against The Arabidopsis Information Resource (TAIR, http://www.arabidopsis.org.gate1.inist.fr) databases were undertaken and unigenes with the highest sequence similarity were functionally annotated using Blast2GO (Conesa et al., 2005) with cut-off e-values of 10e-5 (Blastx) and 10e-6 (mapping). Blast-based annotations were complemented with domain-based annotations using the Inter-ProScan tool (v5). Functional classification of unigenes was based on Blast2GO analysis. Pathway assignments were carried out according to KEGG database (Kyoto Encyclopedia of Genes and Genomes (KEGG) resource; http://www.kegg.jp/ or http://www.genome.jp/kegg/). The statistical assessment of Gene Ontology (GO) term enrichments after xenobiotic treatments in comparison to control condition was performed [Fisher's Exact Test with multiple testing correction; false discovery rate (FDR) < 0.05; *p* < 0.005] as implemented in Blast2GO (Conesa et al., 2005). Comparison of conserved amino acids was performed using Clustal Omega for alignment (Sievers et al., 2011).

### Differential expression analysis

In order to determine differentially expressed (DE) genes between the 4 conditions, the DESeq method was used. This *in silico* normalization method is included in the DESeq Bioconductor package (Anders et al., 2013). As each condition was represented by a single sample of pooled individuals, the differential expression for each gene was estimated by using the variance of expression for this gene across the 4 conditions. Unigenes were considered as DE at *p* < 0.05.

### qRT-PCR validation

Quantitative RT-PCR was used to confirm *in silico* differential expression and analyse the expression of genes potentially involved in chemical stress response in *L. perenne*. qRT-PCR experiments were carried out using, for each condition, five new independent biological replicates of pooled aerial parts (50 mg fresh weight) from 10 plantlets harvested after transfer experiments. RNA from aerial parts was extracted using TRI Reagent® (Sigma) with an additional DNase treatment. RNA was used for cDNA synthesis (Iscript™ cDNA Synthesis kit, Bio-Rad, Hercules, CA, USA). Resulting cDNAs were used to determine expression profiles according to the different treatments. Quantitative PCR was performed using iQ™ SYBR Green Supermix (Bio-Rad, Hercules, CA, USA). Conditions were as follows: 95°C 3 min, and 40 (95°C 15 s, 60°C 30 s, 72°C 30 s) cycles. All samples were run in duplicate for each primer set. Specific primers for each gene selected for analysis were designed according to 454 sequences using Primer3 software (Rozen and Skaletsky, 2000; Supplemental Table 1). The results of the analysis were treated with Gene Expression version 1.1 software. Relative expressions were assessed in relation to the stable expression level of the GAPDH housekeeping gene (Furet et al., 2012).

### Metabolic profiling

Five new independent biological replicates of aerial part samples, each consisting in 10 pooled plantlets, were harvested after a transfer experiment, freeze-dried and used for metabolomic profiling. Samples were extracted and analyzed using gas chromatography mass spectrometry (GC/MS) as described by Serra et al. (2015). Metabolite levels were quantified using XCalibur v2.0.7 software (Thermo Fisher Scientific Inc., Waltham, MA, USA) and expressed as nmol.mg^−1^ of dry weight (DW).

### Statistical analysis

Metabolic parameters were measured on at least five independent replicates of at least 10 individual plantlets. Gene expression quantification was carried out on at least five other independent biological replicates of 10 pooled plantlets. Statistical analyses were carried out with version 3.0.1 of R software, analysis between means was carried out using the non-parametric Mann-Whitney-Wilcoxon test.

## Results

### *De novo* sequence assembly of the *L. perenne* shoot reference transcriptome

A *de novo* transcriptome analysis of *L. perenne* was carried out on leaves from plants subjected to a transfer experiment involving NOAE levels of chemical stressors and short periods of exposure. *L. perenne* seedlings were submitted to 3 conditions of short-term low-level xenobiotic exposure [glyphosate (G), tebuconazole (T), and a combination of the two (GT)] in comparison to control condition (C). Leaf cDNA libraries, corresponding to these 4 conditions, were prepared and sequenced using 454 mass sequencing. Before preprocessing, sequencing of cDNA libraries resulted in approximately 0.7 Gbp of sequence data with a GC content of 54%. The genome size of *L. perenne* is estimated to be 2.7 Gb (Fiil et al., 2011). Each quarter plate run was preprocessed, and the runs of each treatment were pooled, thus leading to 300,795, 249,254, 338,011, and 307,033 high-quality reads for, respectively, control, glyphosate, tebuconazole and glyphosate + tebuconazole treatments (Table 1). The reference transcriptome, which was derived from the complete pool of reads from the 4 conditions (C, G, T, GT), contained 1,195,093 reads (Table 1).

**Table 1:** **Summary of 454 sequencing data**.

**Number of high-quality (HQ) reads**		**Control**	**Glyphosate**	**Tebuconazole**	**Glyphosate + Tebuconazole**
Sequencing reads before preprocessing (Average bp length of HQ reads)	Run 1	224,814 (481 ± 184)	207,483 (450 ± 180)	234,687 (488 ± 176)	227,001 (483 ± 183)
	Run 2	171,316 (401 ± 232)	143,737 (351 ± 218)	193,881 (431 ± 224)	173,328 (411 ± 233)
Reads after 250–1000 bp trimming	Run 1	190,551	169,605	202,685	193,374
	Run 2	112,834	84,325	139,427	116,918
Reads after trimming and preprocessing (poly-A/T, N, Quality)	Run 1	189,143	167,578	200,697	191,833
	Run 2	111,652	81,676	137,314	115,200
Total HQ reads in pools of run 1 and run 2	Pool	300,795	249,254	338,011	307,033
Reference transcriptome	Pool	1,195,093 reads			

Read assembly resulted in an initial reference transcriptome of 14,492 contigs with length ranging from 250 to 6437 bp (Table 2A) and an average contig size of 890 bp. The unmapped reads (172,634 reads) of 250–1000 bp length range were used for a second round of assembly, which resulted in 3564 new contigs. Among the 172,634 reads used, 42,351 remained unmapped and were defined as resulting singletons. Among the 3564 new contigs, 3245 were very similar, if not identical, to contigs built in the first assembly. The 42,351 singletons and the 319 new contigs were added to the 14,492 contigs leading to 57,162 sequences or unigenes (Table 2B) which were used further for whole transcriptome annotation.

**Table 2:** **Assembly results**.

	**Reference**	**Control**	**Glyphosate**	**Tebuconazole**	**Glyphosate + Tebuconazole**
**(A) FIRST ASSEMBLY**					
Number of contigs	14,492	10,155	5773	9109	9408
Total length of contigs (bp)	12,905,107	10,070,689	6,098,760	9,166,250	9,611,071
Number of contig ≥ 1000 bp	4342	3937	2549	3661	3921
Mean contig length (bp)	890 ± 466	991 ± 498	1056 ± 537	1006 ± 508	1031 ± 503
**(B) FINAL ASSEMBLY**					
First assembly	14,492	8089	3646	7005	7256
Second assembly	319	130	50	94	103
Singletons	42,351	8089	6218	12,553	11,025
Total	57,162	22,087	9914	19,652	18,384

The quality of the assembly was checked by comparing the resulting transcriptome to SRA data of *L. perenne* (43,049 sequences, E-MTAB-1556 in ArrayExpress, Vigeland et al., 2013). A tblastx analysis using an *e* < 10e-6 revealed that 45,962 sequences (13,782 contigs and 32,180 singletons) from the present transcriptome matched similar sequences from the deposited SRA data. The reference transcriptome was also compared to the complete mitochondrial (GenBank: JX999996.1) and chloroplastic (NCBI Reference Sequence: NC_009950.1) genomes of *L. perenne* using tblastx program. The mitochondrial genome contains genes corresponding to 14 tRNA, 3 rRNA and 34 proteins (Islam et al., 2013), among which 27 genes matched with high identities (*e* < 10e-6) with unigenes from the present reference transcriptome. Furthermore, 80 genes from the complete chloroplastic genome, which contains genes encoding 76 unique proteins, 30 tRNAs and four rRNAs (Diekmann et al., 2009), were identical or very close (*e* < 10e-6) to the transcriptome unigenes.

In order to validate further the assembled unigenes, sequence-based alignments were performed against the TAIR (*Arabidopsis thaliana*) database by using the tblastx algorithm (Altschul et al., 1997). According to tblastx data, 55.44% (31,696 unigenes) of the matched sequences showed strong homology with TAIR data (*e* < 10e-50), and 84.9% (48,550 unigenes) of the top hits exhibited lower but significant homology (e-value over 10e-6).

### Functional annotation of the *L. perenne* shoot reference transcriptome

Functional annotation of the reference transcriptome was undertaken using the Blast2GO tool and TAIR database. 9311 (16% of all unigenes) unigenes showed no blast hit in the TAIR (*Arabidopsis thaliana*) database. Among the unigenes yielding blast results, 204 (0.32%) were not mapped and 1296 (2.2%) mapped unigenes were not annotated, thus leading to 46,330 (81%) annotated unigenes. In many cases, multiple GO terms were assigned to the same unigene.

**Figure 1:**
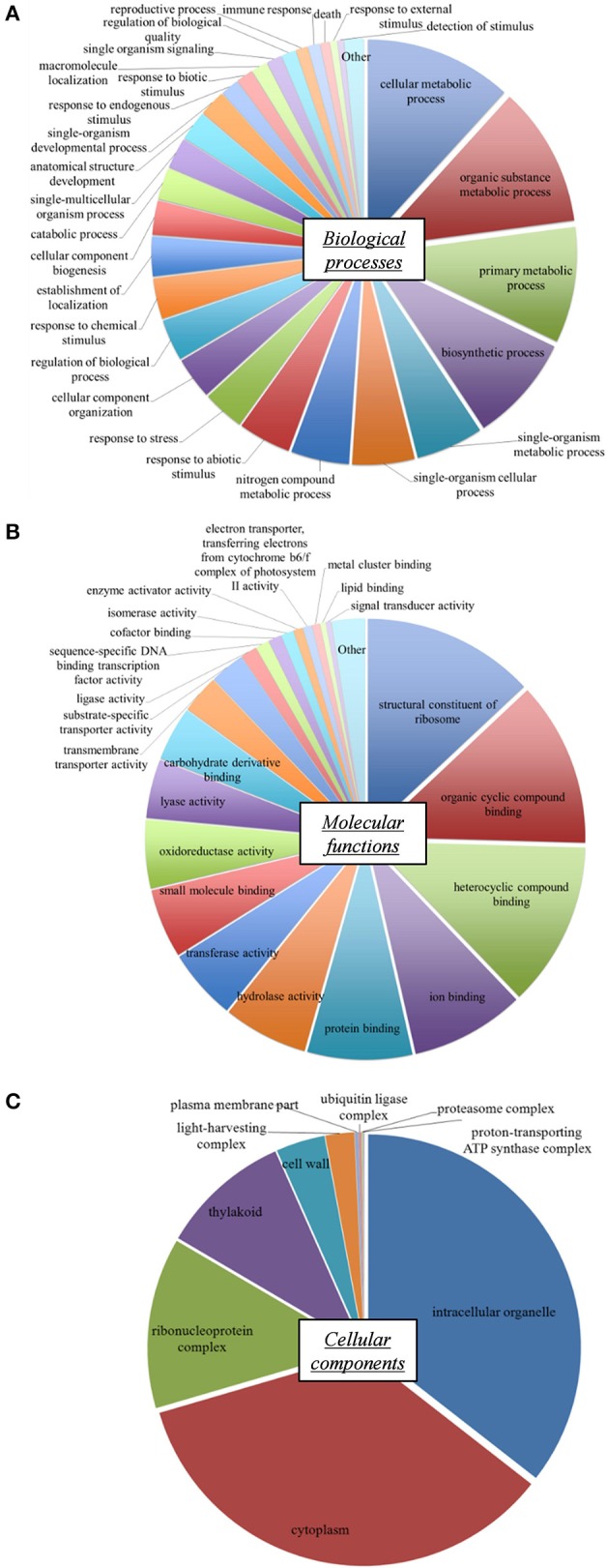
**Distribution of Gene Ontology (GO) terms in the reference shoot transcriptome of ***L. perenne*****. Biological processes **(A)**, Molecular functions **(B)** and Cellular components **(C)** classes are shown. The relative size of pie chart sectors reflects the percentage of annotated unigenes of each GO term category.

GO terms were classified into functional groups according to biological process, molecular function, and cellular component classes. Distribution of the various GO terms identified for the *L. perenne* reference transcriptome is presented in Figure 1 for the major GO classes. The proportions of biological activities were similar to those described in other *L. perenne* transcriptomes (Farrell et al., 2014; Duhoux et al., 2015). GO terms linked to metabolism and biosynthetic processes represented more than 50% of the biological processes (Figure 1A). GO terms related to stress responses, signaling and regulations (response to stress, response to external/abiotic/chemical stimulus, detection of stimulus, single organism signaling, regulation of biological processes) were also well represented (16.6%). Concerning molecular functions (Figure 1B), GO terms linked to various binding activities, as well as activities related to translation (structural constituent of ribosomes), were highly represented. Functional categories also covered enzyme activities such as hydrolase, transferase, oxidoreductase, or lyase activities. The subcellular localization of these processes and functions (Figure 1C) showed the importance of gene products associated with intracellular organelles, chloroplasts (thylakoids, light-harvesting complexes), cytoplasm and ribosomal complexes. Functional distribution therefore demonstrated that this *L. perenne* reference transcriptome exhibited a good coverage of essential types of plant biological activities.

**Figure 2:**
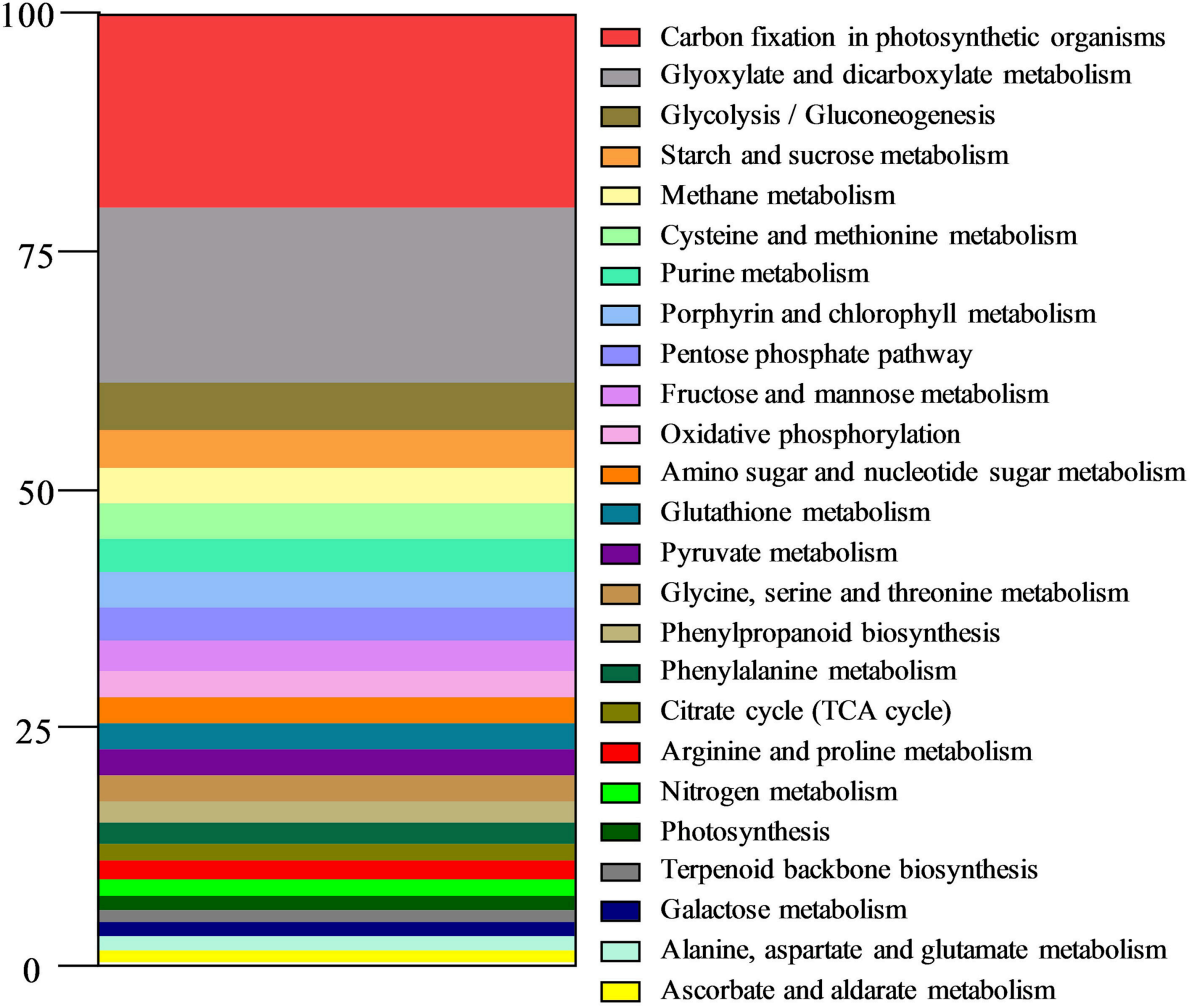
**Distribution of metabolic pathways in the reference shoot transcriptome of ***L. perenne*****. Assignment of annotated unigenes to metabolic pathways was carried out with the KEGG database (Kyoto Encyclopedia of Genes and Genomes (KEGG) resource; http://www.kegg.jp/ or http://www.genome.jp/kegg/). Results are expressed as percentages of annotated unigenes of each metabolic pathway.

The relative importance of various metabolic pathways was assessed from the assignment of unigene annotations in the KEGG database (Figure 2). Most of the predominant pathways were found to belong to carbon metabolism (Glyoxylate and dicarboxylate metabolism, Glycolysis/Gluconeogenesis, Starch and sucrose metabolism, Pentose phosphate pathway, Fructose and mannose metabolism, Amino sugar and nucleotide sugar metabolism, Pyruvate metabolism, TCA cycle, Galactose metabolism, Ascorbate, and aldarate metabolism) and to energy metabolism (Carbon fixation in photosynthetic organisms, Methane metabolism, Oxidative phosphorylation, Nitrogen metabolism, Photosynthesis). Five pathways related to amino acid metabolism were also represented.

### Effects of short-term root-level exposure to NOAE levels of chemical stressors on the relative regulation of functional categories in *L. perenne* leaves

#### Assessment of xenobiotic-related molecular effects

**Figure 3:**
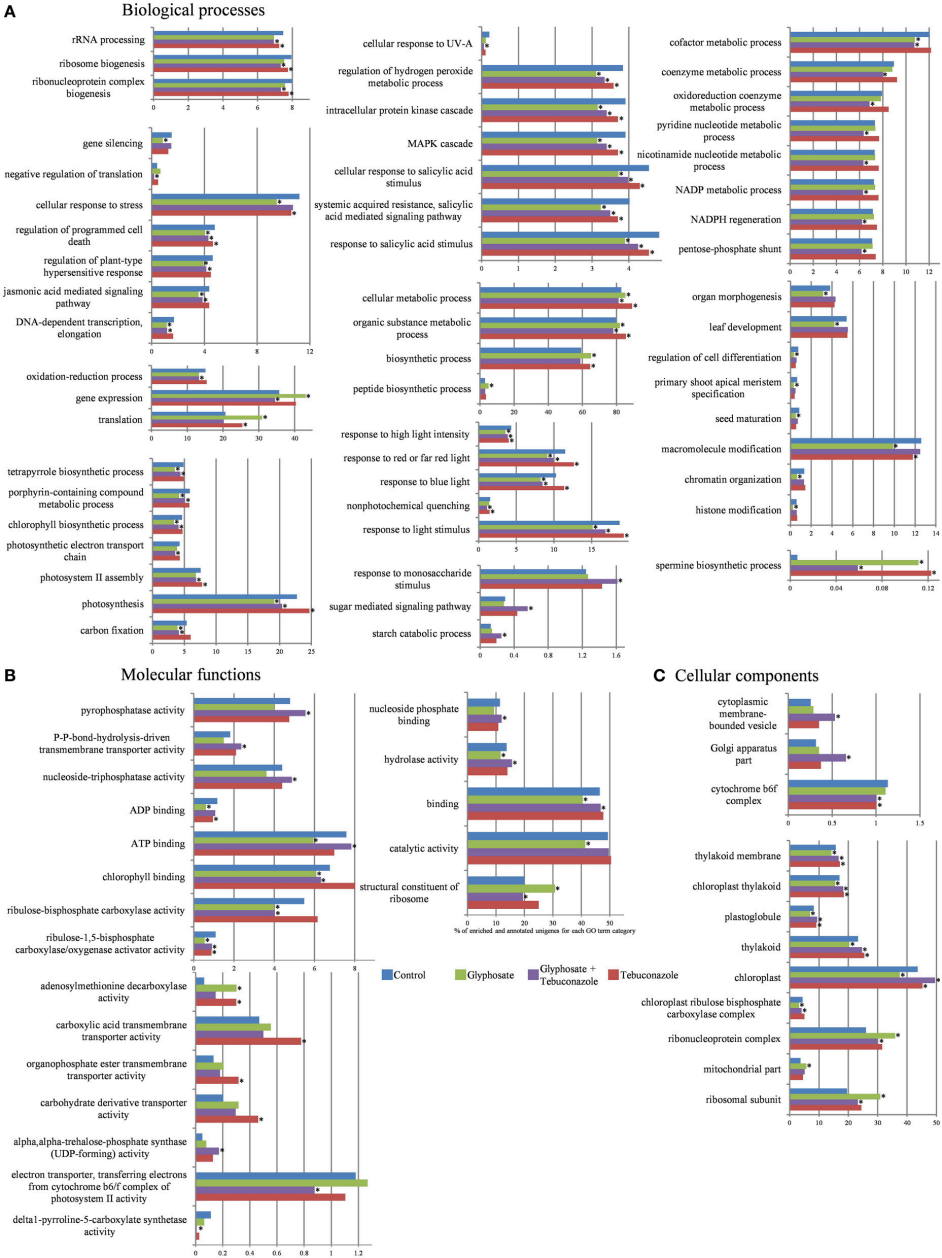
**Effects of xenobiotics on the distribution of representatively-enriched Gene Ontology (GO) terms in the shoot transcriptomes of ***L. perenne*****. Biological processes **(A)**, Molecular functions **(B)**, and Cellular components **(C)** classes are shown. Results are expressed as percentages of enriched and annotated unigenes for each GO term category and for each condition (control, glyphosate, tebuconazole, glyphosate plus tebuconazole) relatively to the total number of annotated unigenes in that class. Stars indicated FDR < 0.05 and *p* < 0.005 after Fisher's Exact Test with robust FDR (false discovery rate) correction.

The relative proportions of functional categories, as defined by GO terms, were analyzed relative to the different conditions of chemical stress. GO enrichment analysis for each class (biological processes, cellular components, and molecular functions) revealed the GO terms for which the quantity of annotated unigenes (contigs and singletons) presented significant differences (FDR < 0.05 and *p* < 0.005) after xenobiotic treatments in comparison to control condition. Results were expressed as the percentage of unigenes in each GO term category in a given condition relative to the total number of annotated unigenes in the same class. The distribution of significantly-enriched GO term categories is given in Figure 3. The percentage of annotated reads in each GO term category in a given condition was also calculated relative to the total number of annotated reads in the same class. In this analysis of annotated read levels, only GO term categories for which at least 50 reads had been counted in at least one condition were selected (Supplemental Table 2). Changes in proportions of GO term categories in terms of unigene enrichment were likely to reflect the effects of xenobiotic treatments on global regulatory processes of genome expression affecting each category. Modifications of proportions of GO term categories in terms of read proportion gave further information on induction or repression.

In a number of cases, the relative proportions of GO term categories were decreased by xenobiotic treatments in terms of percentages relative to annotated unigenes (Figure 3), and also in terms of percentages relatively to annotated reads (Supplemental Table 2). Nevertheless, among all the significant differences induced by treatments, G and T treatments mainly negatively affected the proportion of GO terms relative to control. By contrast, GT treatment induced as much enrichment as depletion in the proportions of GO terms relative to control (Figure 3). Such differences strongly suggested that the mixed GT treatment induced specific effects in comparison with those of single xenobiotic treatments as “response to monosaccharide stimulus”, “sugar mediated signaling pathway”, “starch catabolic process”, “negative regulation of translation”, “pentose phosphate shunt”, “pyrophosphatase activity”, “NADP metabolic process”, “ATP binding”, “Golgi apparatus part”. Moreover G treatment often led to more pronounced effects than those of T and GT treatments (Figure 3).

#### Effects on gene expression and protein translation processes

The “structural constituent of ribosome” category (Figure 3B) showed increased proportions in the presence of G. The potential involvement of ribosome biogenesis in xenobiotic responses was also reflected by the significant increase of “ribosomal subunit” category in the presence of xenobiotics and particularly in presence of G (Figure 3C). Other aspects of the biogenesis of functional ribosomes seemed to be sensitive to chemical stresses, since categories of linked GO terms such as “ribonucleoprotein complex biogenesis”, “rRNA processing” and “ribosome biogenesis” were negatively affected by chemical stress treatments. G and GT treatments also induced a slight decrease of unigene proportion for “DNA-dependent transcription” (Figure 3A). The stability of unigene proportion related to “negative regulation of translation” in the presence of T and G contrasted with a strong decrease of unigene proportion in presence of GT, thus suggesting combination-specific regulations (Figure 3A). For upper-level biological process classes, such as “translation”, overrepresented in presence of G and T, and “gene expression”, overrepresented in presence of G and slightly underrepresented in presence of GT, the increase of unigene proportion in the presence of G (Figure 3A) was associated with an increase in read number (Supplemental Table 2), thus indicating the induction of specific genes. This increase of “gene expression” category occurred in parallel with a decrease in the “gene silencing” category (Figure 3A). Finally, negative effects of G on “histone modification”-related unigenes were observed in upper levels of classification such as “chromatin organization” and “macromolecule modification” (Figure 3A).

#### Effects on photosynthesis and ATP dynamics processes

Unigenes linked to the “ribulose-1,5-bisphosphate carboxylase/oxygenase activator activity” or “Rubisco activator” showed decreased proportions in response to xenobiotics, particularly in response to G (Figure 3B). The associated “ribulose-bisphosphate carboxylase activity” category was significantly more represented in response to T and underrepresented in the presence of G and GT. G and GT treatments reduced unigene proportions corresponding to biological processes associated with “carbon fixation”, “photosynthesis”, “photosystem II assembly”, and “photosynthetic electron transport chain” (Figure 3A). Glyphosate application also reduced the number of unigenes involved in “chlorophyll biosynthetic process” and in related pathways such as “porphyrin-containing compound metabolic process” and “tetrapyrrole biosynthetic process” (Figure 3A). While no unigene enrichment of “magnesium chelatase activity” category was observed, there was an increase in read numbers in the presence of the 3 xenobiotic treatments, for genes whose functions were related to these chlorophyll biosynthetic pathway enzymes (Supplemental Table 2). Xenobiotic-related perturbations in chloroplastic and photosynthetic pathways were also identified for cellular component GO terms (Figure 3C). Numerous unigenes were ascribed to GO terms related to “chloroplast”, “thylakoid”, “plastoglobule”, “chloroplast thylakoid”, and “thylakoid membrane”. These categories generally showed higher proportion of unigenes in the presence of T and GT, while these xenobiotics tended to decrease slightly the proportion of unigenes related to “cytochrome b6f complex” categories (Figure 3C).

The “binding” GO term category was well represented (Figure 3B), in particular with regard to “nucleoside phosphate binding”, “ATP binding”, and “ADP binding”. Proportions of annotated unigenes for these molecular functions decreased in the presence of G, and slightly increased in the presence of GT. However, concerning the “ATP binding” annotation, which was highly represented among GO terms, an increase of the number of corresponding reads was observed in the presence of G, suggesting strong expression of specific and constitutive unigenes (Supplemental Table 2). Likewise, other functions related to nucleoside and nucleotide dynamics, which showed frequent occurrences and high percentages, were responsive to xenobiotic treatments. Thus, the GO term category “nucleoside-triphosphatase activity” showed decreased proportion in the presence of G, whereas GT treatment increased it (Figure 3B). This metabolic activity was characterized by an increase of related read numbers in the presence of G, T, and GT, suggesting strong expression of specific unigenes (Supplemental Table 2). This pattern of modifications was reflected in the changes affecting the corresponding upper-level GO term, “hydrolase activity”. Nucleoside-triphosphatase activity is an essential provider of energy for active transport and is related to the “P-P-bond-hydrolysis-driven transmembrane transporter activity” category, which showed higher proportions of unigenes in the presence of GT (Figure 3B).

#### Effects on environmental response processes

G and GT treatments induced slight decreases of unigene proportions for many biological processes related to abiotic and biotic stress signaling processes, such as “jasmonic acid mediated signaling pathway”, “regulation of plant-type hypersensitive response”, “regulation of programmed cell death”, and “cellular response to stress” (Figure 3A). Moreover, the proportions of unigenes and reads related to “response to salicylic acid”, “MAPK cascade”, “intracellular protein kinase cascade”, and “regulation of hydrogen peroxide metabolic process” were negatively affected by all the chemical stressors (Figure 3A). Most of the biological processes linked to light responses showed a decrease for the proportions of related unigenes in the presence of G and GT. GO term categories such as “response to light stimulus”, and related sublevels such as “non-photochemical quenching”, “response to blue light”, “response to red or far red light”, and “response to high light intensity”, followed the same tendency (Figure 3A).

#### Effects on metabolic functions

The proportions of unigene categories linked to carbohydrates and their derivatives were significantly influenced by chemical treatments. Unigenes annotated in “starch catabolic process”, “sugar mediated signaling pathway”, and “response to monosaccharide stimulus” in biological processes were induced by GT treatments (Figure 3A). GT condition reduced the number of unigenes related to “pentose-phosphate shunt” (Figure 3A). The effects of chemical treatments on the “pentose-phosphate shunt” category were reflected in the responses of closely related categories such as “NADPH regeneration”, “NADP metabolic process”, “nicotinamide nucleotide metabolic process”, “pyridine nucleotide metabolic process”, “oxidoreduction coenzyme metabolic process”, “coenzyme metabolic process”, and “cofactor metabolic process” (Figure 3A).

A number of transport activities in the molecular function category were found to be potentially induced by xenobiotics, particularly by T. This was the case for the “organophosphate ester transmembrane transporter” and “carboxylic acid transmembrane transporter activity” categories (Figure 3B). There was also an increase in the unigene proportion of “cytoplasmic membrane-bound vesicle” category in response to GT (Figure 3C).

Finally, GT treatment decreased the unigene proportion of “delta1-pyrroline-5-carboxylate synthetase (P5CS) activity” (Figure 3B) implicated in the proline biosynthesis pathway.

#### Effects on growth and development processes

All chemical treatments, and more particularly T and G, increased the number of unigenes annotated in the “adenosylmethionine decarboxylase (SAMDC) activity” molecular function category (Figure 3B), which is related to spermidine and spermine biosynthesis from putrescine. In contrast, for the “polyamine oxidase activity” category, which catalyzes the oxidative degradation of polyamines, the related unigenes were not differentially enriched while the number of reads decreased in response to T, G, and GT (Supplemental Table 2). Such maintenance of polyamine levels, which are essential for growth and development in plants (Galston and Sawhney, 1990), may be, at least in part, related to the variations of categories at a higher level of organization. Indeed, T and GT treatments enhanced the number of unigenes correlated to developmental processes such as “seed maturation”, “primary shoot apical meristem specification” and more generally “regulation of cell differentiation”, while G treatment had opposite effects on proportions of unigenes and reads (Figure 3A, Supplemental Table 2). The presence of G also induced a decrease of unigene and read proportions linked to “leaf development” and “organ morphogenesis”.

### Identification and characterization of xenobiotic-responsive genes in *L. perenne*

**Figure 4:**
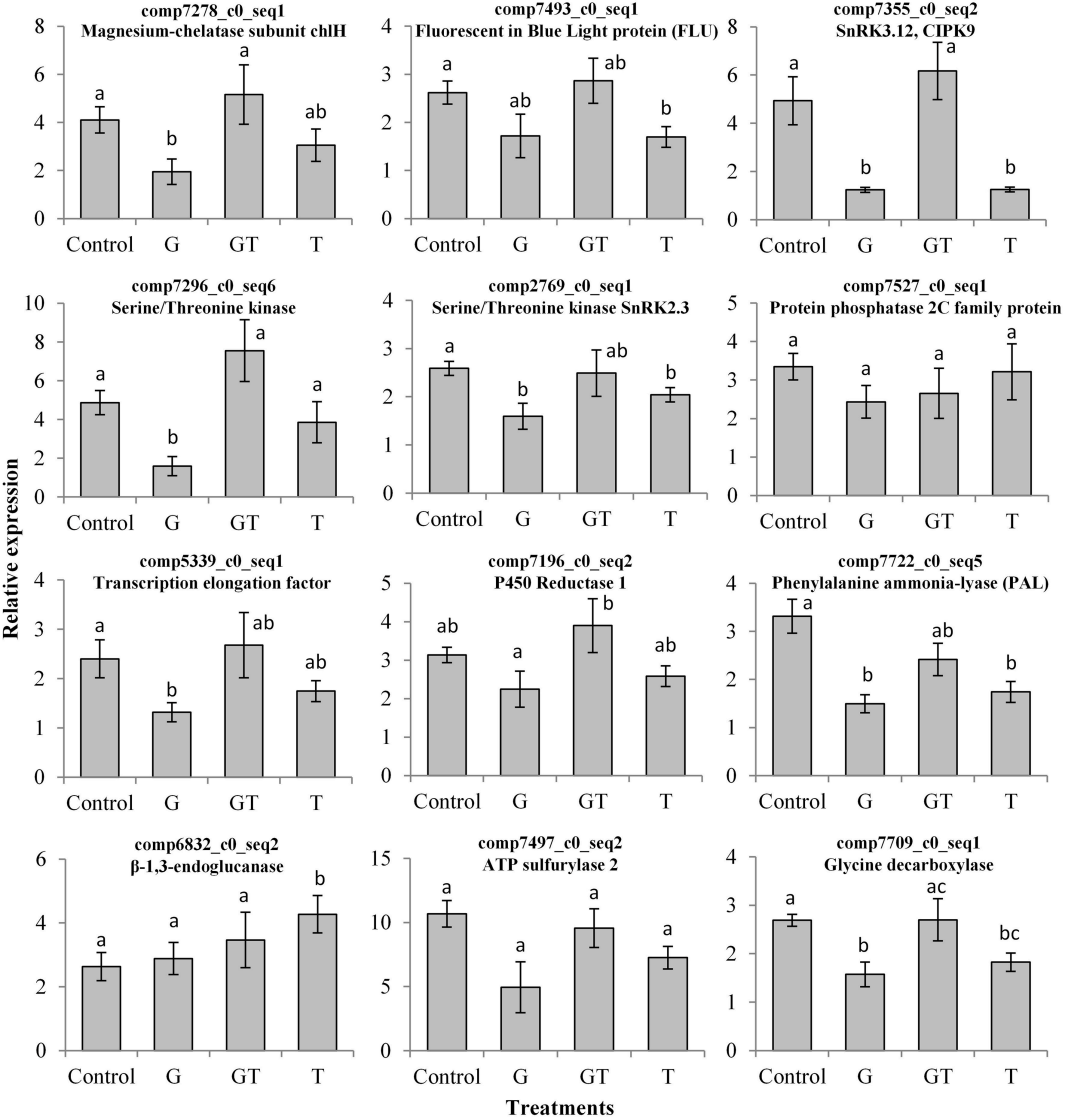
**Effects of xenobiotics on the transcript levels of candidate xenobiotic-responsive genes in leaves of ***L. perenne*****. *L. perenne* plants were grown for 7 days in the absence of pesticide and then transferred to fresh growth medium containing the different xenobiotic treatments [glyphosate (G, 1 μM), tebuconazole (T, 4 μM) and a combination of glyphosate and tebuconazole (GT, 1 μM and 4 μM, respectively)]. Transcript levels (mean ± SEM) were quantified by qRT-PCR after 4 days of treatment. Unigene descriptions were established according to best blast hits in TAIR. Statistical analysis between means was carried out using the Mann-Whitney-Wilcoxon test. Statistical significance of differences (*p* < 0.05) between treatments is indicated by different letters above bars.

Using a *p* < 0.05, DESeq analysis revealed that 69 unigenes were significantly DE in at least one of the four conditions (Table 3). These gene expression profiling data obtained from RNA-seq were complemented with qRT-PCR analysis of the relative expression of candidate genes (Figure 4). The alignments of corresponding protein sequences of candidate unigenes with *Arabidopsis thaliana, Oryza sativa*, and *Brachypodium distachyon* orthologs (Supplemental Figure 1) highlighted high degree of sequence conservation between *L. perenne* and these monocot and dicot model species, and strengthened the automatic *in silico* annotation.

**Table 3:** **Significantly differentially expressed transcripts (DESeq analysis)**.

**Unigene ID**	**Description (TAIR)**	**DEseq analysis Log**_**2**_ **[ratio]**		
		**T/C**	**G/C**	**GT/C**
comp7278_c0_seq1	Magnesium-chelatase subunit chlH	Repressed	Repressed	0.304
comp7493_c0_seq1	Protein FLUORESCENT IN BLUE LIGHT (FLU)	−2.456	Repressed	−1.124
comp7590_c0_seq3	Plastid transcriptionally active (PTAC16)	Induced	Induced	Induced
comp7590_c0_seq4	Plastid transcriptionally active (PTAC16)	Repressed	Repressed	−1.124
comp7427_c0_seq4	Light harvesting complex photosystem II	Repressed	Repressed	−1.149
comp7634_c0_seq2	Photosystem I, PsaA/PsaB protein	0.939	Repressed	0.069
comp7693_c0_seq11	Photosystem II reaction center protein D	3.129	4.874	Repressed
comp7726_c0_seq27	Photosystem II reaction center protein B	−1.363	Repressed	−2.879
comp7726_c0_seq5	Photosystem II reaction center protein B	−1.704	1.167	Repressed
comp7726_c0_seq1	Photosystem II reaction center protein B	−0.434	Repressed	−0.654
comp6560_c0_seq1	Photosystem I P subunit	1.222	Repressed	−1.372
comp6663_c0_seq5	Isoleucyl-tRNA synthetase-like protein	Repressed	Repressed	1.706
comp7674_c0_seq3	Phosphoenolpyruvate carboxylase	Induced	NDE	NDE
comp6943_c0_seq1	Uncharacterized protein	−0.380	Repressed	−0.366
comp6984_c0_seq1	Rubredoxin family protein	Induced	NDE	Induced
comp6746_c0_seq2	Actin 7	Repressed	2.586	3.379
comp7483_c1_seq2	Phosphoglycerate kinase family protein	−0.723	Repressed	−2.239
comp7355_c0_seq2	CIPK9, SnRK3.12, PKS6, CBL-interacting protein kinase	1.097	Repressed	1.794
comp7489_c0_seq2	Calcium sensing receptor	Repressed	2.415	0.654
comp7362_c0_seq6	Aldehyde dehydrogenase 3F1	−2.379	2.737	0.291
comp7713_c0_seq4	Protein kinase superfamily protein	Induced	NDE	Induced
comp7705_c0_seq3	Phototropin serine-threonine kinase	Repressed	Repressed	0.645
comp7527_c0_seq1	Protein phosphatase 2C family protein	2.737	Repressed	−0.516
comp7476_c0_seq2	Ferredoxin-NADP(+)-oxidoreductase 1 (FNR)	−1.100	Repressed	0.670
comp7633_c0_seq4	4-hydroxy-3-methylbut-2-enyl diphosphate synthase	Induced	NDE	Induced
comp7402_c0_seq2	F-type H+-transporting ATPase subunit delta	Repressed	4.265	1.863
comp7682_c1_seq3	H(+)-ATPase 2	Repressed	−1.322	0.192
comp7721_c0_seq19	ATP synthase subunit alpha	Induced	NDE	Induced
comp7721_c0_seq17	ATP synthase subunit alpha	Repressed	1.415	−1.294
comp7700_c0_seq2	ABC transporter F family	−0.214	Repressed	0.016
comp7571_c0_seq5	Major facilitator superfamily protein	0.959	Repressed	0.564
comp7726_c0_seq35	RNA polymerase subunit alpha	Induced	NDE	NDE
comp7697_c0_seq14	Homeodomain-like superfamily protein	−0.029	Repressed	0.232
comp7697_c0_seq11	Homeodomain-like superfamily protein	0.222	Repressed	Repressed
comp7688_c0_seq2	NAD(P)-binding Rossmann-fold superfamily protein	Repressed	0.023	−0.101
comp7592_c0_seq2	Translation initiation factor IF	−0.778	Repressed	1.128
comp6892_c0_seq2	Gametogenesis related family protein	0.824	2.314	0.914
comp7302_c0_seq1	Uncharacterized protein	0.740	1.816	0.545
comp7589_c0_seq4	Chloroplast stem-loop binding protein	3.392	4.265	Repressed
comp7452_c0_seq3	Indoleacetic acid-induced protein	Induced	NDE	Induced
comp7604_c0_seq2	Tubulin alpha-5	−2.100	Repressed	0.429
comp7501_c0_seq5	DNA photolyase	−0.314	Repressed	−0.982
comp7711_c0_seq1	Presequence protease 1	−1.397	−2.426	Repressed
comp7711_c0_seq5	Presequence protease 1	NDE	Induced	Induced
comp7711_c0_seq3	Presequence protease 1	Induced	NDE	Induced
comp7458_c0_seq2	Peroxidase superfamily protein	Induced	NDE	Induced
comp7458_c0_seq1	Peroxidase superfamily protein	Repressed	−0.285	−1.409
comp7628_c1_seq8	Senescence-associated protein	−0.555	1.415	Repressed
comp7309_c0_seq3	Glycolate oxidase 1 (GOX1)	2.410	Repressed	−1.294
comp7324_c0_seq3	Phenylalanine ammonia-lyase	Repressed	0.337	−0.957
comp7722_c0_seq7	Phenylalanine ammonia-lyase	Repressed	1.678	Repressed
comp7722_c0_seq5	Phenylalanine ammonia-lyase	−0.710	−2.977	Repressed
comp7722_c0_seq3	Phenylalanine ammonia-lyase	NDE	NDE	Induced
comp6929_c0_seq4	O-methyltransferase	Repressed	0.093	−1.031
comp7591_c1_seq3	ACC oxidase 5 (ACO5)	Induced	Induced	Induced
comp7591_c1_seq4	ACC oxidase 5 (ACO5)	Repressed	−0.700	Repressed
comp6832_c0_seq2	β-1,3-endoglucanase	2.485	Repressed	0.969
comp7382_c0_seq3	Glutamate synthase 1 [NADH]	0.753	Repressed	−0.294
comp7386_c0_seq10	Polyamine oxidase	Induced	NDE	Induced
comp7536_c0_seq5	Sulfite reductase	Induced	NDE	Induced
comp7497_c0_seq1	ATP sulfurylase 2	Repressed	1.830	−0.071
comp7497_c0_seq2	ATP sulfurylase 2	0.222	Repressed	Repressed
comp6892_c0_seq1	Uncharacterized protein	0.850	2.148	0.842
comp7454_c0_seq1	Uncharacterized protein	−0.915	1.126	Repressed
comp7706_c0_seq8	Dentin sialophosphoprotein-related	Repressed	0.699	Repressed
comp7528_c0_seq1	Uncharacterized protein	2.170	1.608	−0.346
comp7706_c0_seq6	Dentin sialophosphoprotein-related	Induced	Induced	Induced
comp7188_c0_seq3	Unknown protein	Induced	Induced	Induced
comp6657_c0_seq2	Pentapeptide repeat-containing protein	Induced	Induced	Induced

*C, control; G, glyphosate; T, tebuconazole; GT, glyphosate + tebuconazole. The log_2_ [ratio] values could not be calculated when no expression was detected for a given transcript. Such cases were indicated by “Induced” when no expression was detected in the control, “Repressed” when no expression was detected in the treatment conditions. Unigenes were considered as differentially expressed at p < 0.05. “NDE” indicated cases for which transcripts were non-differentially expressed. Unigene descriptions were established according to best blast hits in TAIR. The intensities of red or green colors respectively indicate increase or decrease of expression levels in the presence of G, T, and GT compared to the control condition*.

Most of the observed differential expressions (Table 3) validated the analysis of GO term category variations under conditions of xenobiotic treatment (Figure 3). Among the DE unigenes, a high proportion of xenobiotic-repressed genes were associated with photosynthesis or chlorophyll biosynthetic processes. This was particularly the case for the transcripts of genes related to the tetrapyrrole pathway, such as those encoding Magnesium-chelatase subunit chlH and Fluorescent In Blue Light (FLU), a tetratricopeptide repeat (TPR)-containing protein, highly conserved between *A. thaliana, O. sativa*, and *B. distachyon* (Supplemental Figure 1) that were decreased by xenobiotic treatments (Table 3). This decrease was confirmed by qRT-PCR, when glyphosate or tebuconazole were applied alone (Figure 4). Similar expression profiles were observed for all photosynthesis- and chlorophyll-related unigenes, except for one isoform encoding the plastid transcriptionally active 16 (*PTAC16*) gene that was induced by all conditions (Table 3). Induction of expression under G and T treatments was detected by DESeq for the transcript annotated “photosystem II reaction centre protein D”. This was also the case for some genes related to photosystems, which exhibited significant induction under either G or T treatments (Table 3).

The DESeq analysis revealed that a high proportion of xenobiotic-responsive genes are involved in signaling, and more particularly, correspond to signal transduction through protein-kinases and protein-phosphatases (Table 3). This was the case for the CIPK9-annotated gene, which encodes a Calcineurin B-Like Protein (CBL)-interacting protein kinase highly conserved between monocot and dicot species (Supplemental Figure 1). This Serine/Threonine protein kinase annotated unigene, also known as sucrose non-fermenting (SNF)-related kinase family of serine/threonine kinase 3.12 (SNRK3.12), as well as a calcium sensing receptor-annotated transcript, were found to be DE (Table 3). DESeq showed a repression of *CIPK9* by G and an induction by T and GT (Table 3). qRT-PCR analysis confirmed this repression by G (Figure 4).

Transcripts related to protein phosphorylation and dephosphorylation were highly represented in the different datasets. The effects of xenobiotic stresses on highly conserved Serine/Threonine protein kinases (Supplemental Figure 1), such as comp7296_c0_seq6, annotated as a Serine/Threonine protein kinase, and a unigene comp2769_c0_seq1, annotated as a SNRK2 protein (Kertesz et al., 2002), were analyzed by qRT-PCR (Figure 4). Glyphosate, like for the CIPK9-annotated gene, repressed expression of these genes (Figure 4). As observed for SnRK2-type unigenes, glyphosate tended to repress a PP2C-annotated gene, comp7527_c0_seq1 (Table 3, Figure 4). Different types of expression profile were found, such as the high upregulation of F-type H^+^-transporting ATPase subunit delta-encoding gene by G, the specific repression of the unigene related to H^+^-ATPase 2 by G or T, or the induction by T or GT of one of the isoforms encoding an ATP synthase subunit alpha (Table 3).

The expression of unigenes associated with the regulation of translation or transcription also differed depending on the chemical stressor. Glyphosate repressed the expression of genes associated with Homeodomain-like superfamily proteins and involved in the regulation of transcription (Table 3). Another transcription elongation factor encoded by comp5339_c0_seq1 presented the same kind of expression profile (Figure 4). On the contrary, the expression of 3 unigenes, whose function was linked to translation (comp6892_c0_seq2, comp7302_c0_seq1, and comp7589_c0_seq4), was significantly upregulated by glyphosate and GT (Table 3). A *GOX1* gene encoding a Glycolate oxidase was differentially repressed in the presence of glyphosate and induced in presence of tebuconazole (Table 3). Genes encoding Cytochrome P450 reductase proteins tended to be repressed by glyphosate, whereas the GT mixture had opposite effects (Figure 4).

The qRT-PCR analysis of one of genes annotated as Phenylalanine ammonia-lyases (PAL) demonstrated the repressive effect of xenobiotics on its expression (Figure 4). A gene homologous to a gene encoding a caffeate *O*-methyltransferase was repressed by T and GT (Table 3). 1-aminocyclopropane-1-carboxylic acid (ACC) oxidases were characterized in the shoot transcriptome of *L. perenne* by two isoforms that were differentially regulated, with one being induced by chemical stresses, while the other was repressed (Table 3). In parallel, the gene annotated as β-1,3-endoglucanase was induced by T and GT, as confirmed by qRT-PCR trends for T treatment (Table 3, Figure 4). The sulfur assimilation pathway, which leads to cysteine biosynthesis and *in fine* to glutathione (GSH) biosynthesis, involves, *inter alia*, ATP sulfurylase and sulfite reductase (Anjum et al., 2015). In *L. perenne*, one of the 2 isoforms annotated as ATP sulfurylase showed repression by T and induction by G, while the other was repressed by G and GT (Table 3). The qRT-PCR analysis also showed that G tended to repress this latter isoform (Figure 4). In contrast, T and GT had a positive impact on the transcription of the sulfite reductase-annotated gene (Table 3). Finally, differential expression analysis revealed several uncharacterized transcripts whose expression, for most of them, was induced in the presence of glyphosate and with contrasting effects of the other treatments (Table 3).

### Metabolic modifications in *L. perenne* leaves under conditions of short-term root-level exposure to NOAE levels of chemical stressors

**Figure 5:**
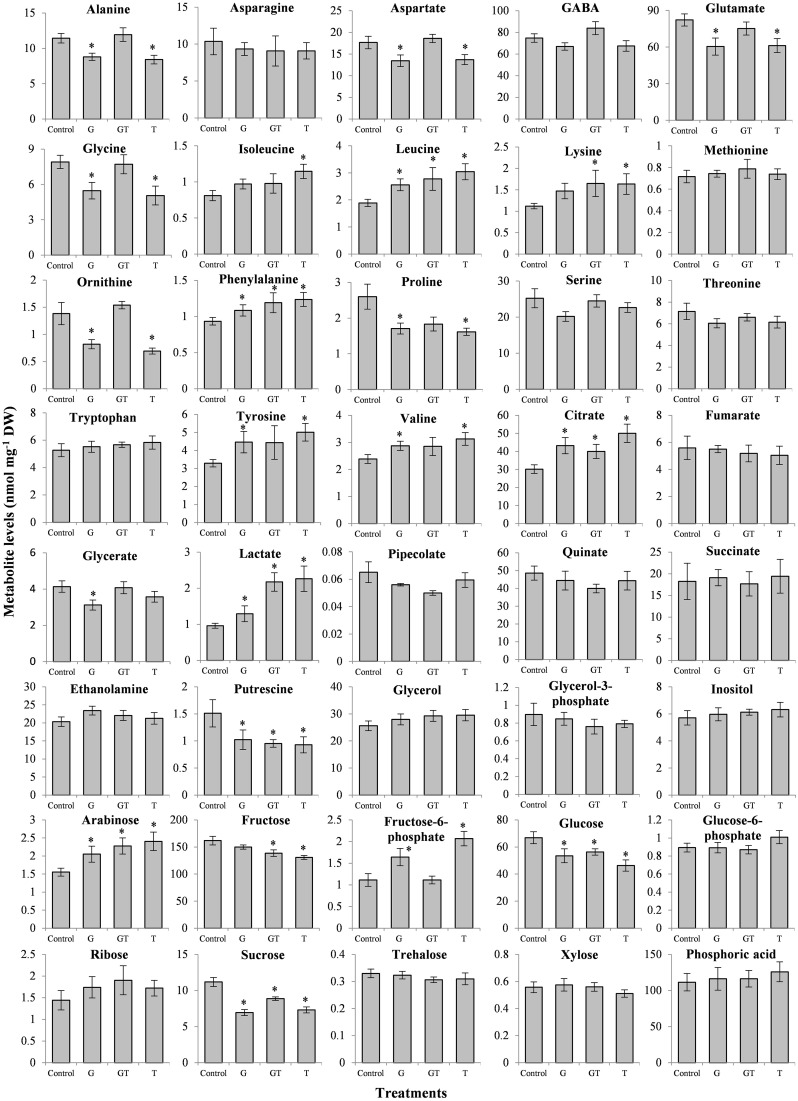
**Effects of xenobiotics on metabolite levels in ***L. perenne*** leaves**. *L. perenne* plants were grown for 7 days in the absence of pesticide and then transferred to fresh growth medium containing the different xenobiotic treatments [glyphosate (G, 1 μM), tebuconazole (T, 4 μM) and a combination of glyphosate and tebuconazole (GT, 1 μM and 4 μM, respectively)]. Metabolite levels (mean value ± SEM) were quantified after 4 days of treatment. Stars indicated statistical significance of differences between treatment and Control (*p* < 0.05) carried out using the Mann-Whitney-Wilcoxon test.

The effects of the present xenobiotic stress conditions (low intensity, short-term exposure, root exposure) were also analyzed in terms of metabolic changes in *L. perenne* leaves (Figure 5). Moreover, as previously described in *A. thaliana* (Serra et al., 2013), major metabolic changes were found to occur despite the absence of major stress perturbations. All of the subtoxic conditions analyzed here (G, T, GT) induced or tended to induce a decrease of soluble sugar contents (Fru, Glc, Suc) whereas Tre levels were no affected (Figure 5). In contrast, G and T acted differently on the levels of Fru-6-P compared to the GT mixture, with an increase of this phosphorylated sugar with G and T and no effect with GT. Glc-6-P remained relatively stable in response to the three conditions.

Some metabolic changes could be related to the patterns of differential expression described above (Figures 3, 4, Table 3). Glyphosate or tebuconazole exposure induced a decrease of glycine levels (Figure 5) and also affected the expression of glycine-related genes such as glycine decarboxylase (Figure 4). The decrease in putrescine levels in *L. perenne* leaves in response to xenobiotic treatments (Figure 5) was in line with the induction by T and GT of the unigene encoding a polyamine oxidase (Table 3) and with significant variations of related GO term categories, as “adenosylmethionine decarboxylase (SAMDC) activity” and “spermine biosynthetic process” (Figure 3). The decrease of glutamate levels in *L. perenne* leaves in response to G (Figure 5) was reminiscent of the decrease of glutamate synthase expression (Table 3).

All treatments tended to increase the levels of arabinose, Phe, Lys, Leu, Ile, Val, Tyr, citrate and lactate and to decrease the levels of putrescine and Pro. In particular, glyphosate treatment did not decrease levels of aromatic amino acids (Trp, Tyr, Phe). For Ala, Asp, Fru-6-P, glutamate and ornithine, effects of G or T alone were mostly comparable, and GT mixture effects were not additive (Figure 5). In the case of stress metabolites, pipecolate, putrescine, inositol and Pro decreased or were not affected, while lactate increased by low chemical stress (Figure 5). This decrease of Pro levels of plants subjected to GT (Figure 5) could be related to the reduction of proportion of genes related to “P5CS activity” category (Figure 3B).

## Discussion

### Xenobiotic-regulated RNA-seq and qRT-PCR markers reveal novel potential pathways of chemical stress responses

**Figure 6:**
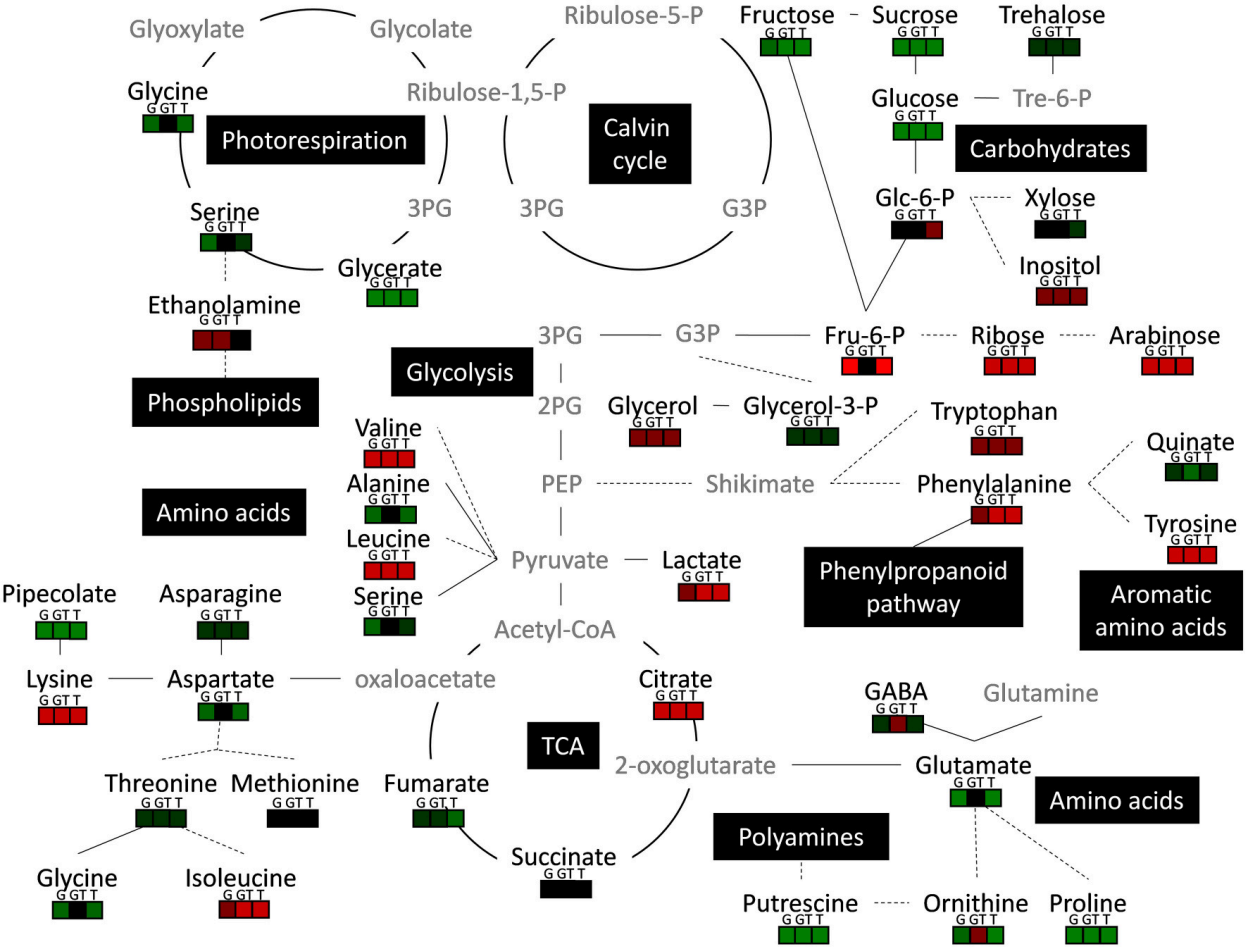
**Metabolic integration of low-intensity xenobiotic stress in ***L. perenne*****. Black names represent quantified metabolite and gray names represent non-quantified metabolites. 2PG, 2-Phosphoglycerate; 3PG, 3-Phosphoglycerate; APS, adenosine 5′-phosphosulfate; G3P, glyceraldehyde-3-phosphate; PEP, Phosphoenolpyruvate; SAMDC, S-adenosylmethionine decarboxylase; Spd, spermidine; Spm, spermine. The red or green colors respectively indicate increase or decrease of metabolite levels in the presence of glyphosate (G), tebuconazole (T) and a combination of glyphosate and tebuconazole (GT) compared to the control condition. Solid line indicate direct relation between metabolites, and dotted line show indirect reaction.

The present *de novo* RNA-Seq approach yielded a *L. perenne* xenobiotic-dependent genomic database (Figures 1, 2) that was coherent with recent transcriptomic analyses of *L. perenne* (Farrell et al., 2014; Duhoux et al., 2015) and that was very useful to explore relationships between metabolic changes, stress adjustments, regulation of metabolism genes and signaling pathways under conditions of xenobiotic stress. The experimental conditions of short-term exposure and low levels of xenobiotics led, in all of the cases (glyphosate, tebuconazole, GT mixture), to no observable adverse effects, especially on root growth, which is particularly xenobiotic-sensitive (Serra et al., 2013, 2015). However, these conditions were sufficient to induce significant molecular and metabolic changes (Figures 3–6), which could be expected to reflect pre-damage, and probably primary events in the xenobiotic response. The strong impact of these various xenobiotic treatments on the proportions of transcript functional categories and on individual gene expression established that low-level xenobiotic exposure strongly interacted with molecular regulations of gene expression. Moreover, differential effects of the different xenobiotic treatments showed that there were some xenobiotic-dependent specificities in the potential molecular mechanisms of xenobiotic action and of plant responses to xenobiotics, and also that there were specific responses for mixture (Figures 3–5). However, this global analysis also revealed the involvement of common mechanisms that are interdependent with each other and closely connected. Finally, detection of major metabolic and molecular rearrangements in leaves, which were not the primary site of exposure, demonstrated that root exposure involved strong xenobiotic-related root-shoot communication, through xenobiotic, metabolite or signal transport, underlining the impact of such exposure.

Major genes that were found to be DE under xenobiotic exposure did not belong to classical xenobiotic or herbicide stress response pathways such as detoxification pathways involving glutathione S-transferase, cytochrome P450 or glycosyltransferase enzyme activities (Duhoux and Délye, 2013; Délye, 2013), thus suggesting that novel molecular mechanisms leading to xenobiotic-induced metabolic rearrangements have been identified. Most of the 69 DE genes, underlined without *a priori* by the DESeq analysis, surprisingly formed a coherent set as shown by the several associated pathways described below. A significant number of xenobiotic-regulated genes were linked to signal metabolisms and to signal transduction pathways, thus underlining the importance of homeostatic mechanisms and crosstalks between metabolites, carbohydrates, phytohormones, plastid-to-nucleus and light signaling under conditions of xenobiotic stress. The DESeq analysis also suggested that activities of phosphorylation linked to signal transduction could be affected by differential expression of genes related to MAPK cascade and to ATP synthesis or hydrolysis (Table 3). Increase of phenylalanine levels in leaves in response to GT and T treatments was associated with repression of genes related to phenylalanine ammonia lyase (PAL) activity (Figures 4–6). PALs catalyze the first step of the phenylpropanoid pathway and have overlapping roles in plant growth, development, and responses to environmental stresses (Huang et al., 2010). When added at high and toxic levels, the herbicides paraquat and glyphosate induce PAL activity in plants, thus suggesting involvement of PAL in regulation of the phenylpropanoid pathway in response to oxidative stress produced by lethal herbicide levels (Duke et al., 1980; Lee et al., 2003). In *L. perenne*, low-intensity xenobiotic stress also had significant gene expression effects (Table 3, Figure 4) on the gene annotated as Cytochrome P450 reductase protein, which functions in electron transfer from NADPH to cytochrome P450 oxidases, and on a caffeate *O*-methyltransferase-homologous gene. These enzyme activities may be both involved in the general phenylpropanoid pathway, as described by Sundin et al. (2014) and Do et al. (2007) for Arabidopsis, strengthening the involvement of PAL in chemical stress responses. PAL, ACC synthase, which catalyzes the synthesis of ACC for ethylene production, and auxin biosynthesis are thought to be co-regulated (Duke et al., 1980; Soeno et al., 2010). Tebuconazole treatment increased the expression of the gene annotated as β-1,3-endoglucanase and of an ACC-oxidase-annotated gene (Table 3), which functions are both related to ethylene action through regulation or metabolism (Zhong and Burns, 2003; Soeno et al., 2010; Jafari et al., 2013). These molecular markers confirmed the relationships between xenobiotic effects, phytohormone, and more particularly ethylene, dynamics and signaling, as previously reported in studies coupling hormone-signaling mutants and xenobiotic-induced stress (Sulmon et al., 2007; Weisman et al., 2010).

GO term enrichments allowed to detect by reads counting the overexpression of unigenes whose functions were related to “magnesium chelatase activity” (Supplemental Table 2). Nevertheless, comp7278_c0_seq1, whose direct function, according to its annotation, is a magnesium chelatase activity (Magnesium-chelatase subunit ChlH), presented an expression significantly affected by glyphosate and tebuconazole (Table 3, Figure 4), suggesting that overexpressed unigenes may be involved in the negative regulation of expression of the magnesium chelatase activity. The Fluorescent In Blue Light (FLU)-annotated gene was also significantly affected by glyphosate and tebuconazole single treatments (Table 3, Figure 4). FLU and ChlH are involved in regulation of tetrapyrrole biosynthesis (Kauss et al., 2012; Apitz et al., 2014) and in one of the multiple signaling pathways which mediate plastid signals controlling expression of Photosynthesis-Associated Nuclear Genes (PhANG; Mochizuki et al., 2008; Moulin et al., 2008; Zhang et al., 2013). Moreover, tetrapyrrole gene expression and ABA signaling are interconnected in chloroplast-to-nucleus signaling pathways (Voigt et al., 2010). Zhang et al. (2011) showed that resistance to concomitant high-light and herbicide stress requires the activity of ABI4, an ABA-regulated Apetala 2 (AP2)-type transcription factor, which is also involved in the coordination of several pathways such as sugar, redox, and hormonal [ABA, jasmonate (JA) and salicylic acid] signaling during stress response (Foyer et al., 2012). ABA catabolism markers were also reported to be affected in response to chemical stress treatments as glyphosate (Serra et al., 2013) and triazole including tebuconazole (Saito et al., 2006; Serra et al., 2013). Moreover, triazoles have also been shown to be inhibitors of brassinosteroid synthesis (Kaschani and van der Hoorn, 2007), of the plant sterol pathway in Sorghum (Lamb et al., 2001) and of gibberellin accumulation in rapeseed (Child et al., 1993). Thus, different hormone pathways and signaling seem to be important in numerous xenobiotic responses (Couée et al., 2013). As an example, promoter-level induction by glyphosate and hormones has been described in rice for a glutathione S-transferase involved in xenobiotic detoxification (Hu et al., 2011). However, further experimental studies need to be carried out in order to clearly establish the role of these hormones in plant chemical stress responses.

### Low levels of xenobiotics have significant effects on multiple low energy and low carbohydrate regulations

Genes related to components of photosynthetic reaction centers presented patterns of expression that were largely influenced by xenobiotics. The gene annotated as photosystem II reaction center protein D presented the highest observed induction by G and T and repression by GT. More generally, differential regulation of genes related to photosynthesis in the presence of sub-lethal concentrations of xenobiotics was specific of the xenobiotic involved (Table 3). Some herbicides, such as atrazine, act through binding to D1 protein of photosystem II (PSII) reaction center, thus blocking electron transfer to the plastoquinone pool (Rutherford and Krieger-Liszkay, 2001; Sulmon et al., 2004). However, neither glyphosate nor tebuconazole are known to affect photosynthesis through this mechanism of action. These variations may thus originate from non-target effects, in accordance with the maintenance of aromatic amino acid levels under G treatment (Figure 5), suggesting that the canonical EPSPS target of glyphosate was not affected. These differential regulations of photosynthesis-related genes by chemical treatments did not however result in modifications of photosynthetic parameters (Serra et al., 2015), as also reported by Ramel et al. (2007) and Faus et al. (2015), underlining adjustment processes to maintain, at least temporarily, efficient carbon assimilation.

Variations of carbohydrate-related and energy-related GO term categories (Figure 3) and, more directly, the variations of soluble sugars (Figure 5) strongly indicated that carbohydrate and energy metabolisms were involved in the responses to low-intensity xenobiotic stress. The level of water-soluble carbohydrates is particularly important as a primary source of energy in *L. perenne* (Smith et al., 1998). Some of the metabolic changes induced by xenobiotics, especially soluble sugar depletion and lactate accumulation (Figures 5, 6), therefore suggested a situation of energy imbalance. All of this strongly suggested that low-intensity xenobiotic stress could be perceived as a central energy perturbation. It must be highlighted that such a relationship has been described in other plant-xenobiotic experimental systems (Qian et al., 2014; Serra et al., 2015). Stress-induced energy deficit typically leads to the activation of genes involved in catabolism (proteolysis, amino acid catabolism, sugar degradation, lipid mobilization) and repression of genes involved in protein synthesis (Valluru and Van den Ende, 2011; Ramel et al., 2012; Serra et al., 2013). The strong variations of amino acid levels (Figure 5) and of amino acid-related genes (Figure 4, Table 3) may reflect the mobilization of amino acid substrates (Valluru and Van den Ende, 2011; Ramel et al., 2012; Serra et al., 2013; Duhoux et al., 2015). Further studies are needed to characterize the relations between amino acid and carbohydrate metabolisms, energy perturbation and this particular NOAE situation produced by short exposure to low doses of xenobiotics.

The potential variations of spermine and spermidine levels, that could be deduced from the strong increase in unigenes related to the “spermine biosynthetic process” category (Figure 3A), and from the decrease of putrescine levels in shoots from xenobiotic-treated plants (Figure 5), also suggested that low-level xenobiotic exposure caused rearrangements of N-related metabolisms.

Situations of low carbohydrate and low energy can be strongly associated with oxidative stress processes (Couée et al., 2006; Baena-González et al., 2007; Dietrich et al., 2011; Valluru and Van den Ende, 2011). The differential expression of genes linked to the pentose phosphate shunt (Table 3) could thus be related to variations of carbohydrate levels and to the supply of reducing equivalents for antioxidant defenses (Couée et al., 2006). Low-intensity xenobiotic stress modified the expression of other genes related to ROS defense. A Glycolate oxidase (GOX1)-annotated gene was found to be regulated in a contrasted manner by glyphosate, tebuconazole and GT treatments (Table 3). Glycolate oxidase is involved in the oxidation of glycolate leading to the production of glyoxylate and H_2_O_2_ during photorespiration (Fahnenstich et al., 2008). In parallel, the levels of photorespiration metabolites (Figures 5, 6) and the expression of a Glycine Decarboxylase-annotated gene (Figure 4) were strongly affected by xenobiotic treatments. These results suggested that these conditions of mild chemical stress, and in relation with carbohydrate, energy and ROS processes, could affect photorespiration.

### Various molecular markers point out to the importance of SnRKs in xenobiotic stress responses

Sugar and energy limitation induced by abiotic stresses are regulated in plants by an array of metabolic sensors (Baena-González and Sheen, 2008; Dietrich et al., 2011). One of these sensors, SnRK1, is involved in responses to a range of stresses that limit photosynthesis and respiration, including the PSII-inhibiting herbicide, DCMU (Baena-González et al., 2007). Sugars, and more particularly Glc-6-P produced by hexokinase (HXK) and Tre-6-P, can block SnRK1 activity (Baena-González and Sheen, 2008; Ramon et al., 2008; Valluru and Van den Ende, 2011; Nunes et al., 2013). Our previous global analysis in *L. perenne*, combining leaves and root metabolites after short exposure and direct and long exposure, showed that various xenobiotic treatments mostly increased Glc-6-P and its correlated co-metabolite, Fru-6-P, levels (Serra et al., 2015). Under the present conditions of short exposure, Glc-6-P remained relatively stable in leaves, suggesting that SnRK1 may not be inhibited by the phosphorylated sugar.

In the present case of *L. perenne*-xenobiotic interactions, several genes, annotated as F-type H^+^-transporting ATPase, H^+^-ATPase, and ATP synthase, were DE in the presence of xenobiotics (Table 3). Moreover, repression of a gene potentially involved in ATP hydrolysis and induction of ATP synthesis genes suggested some kind of cellular mobilization for energy restoration under conditions of xenobiotic exposure (Table 3). Decrease of, or threat to, ATP levels occurring under environmental stresses, including xenobiotic stress, were also related to the involvement of mammalian homolog of SnRK1, the AMP-activated kinase (AMPK) energy sensor (Blättler et al., 2007; Hardie et al., 2012).

SnRK1 and several other serine/threonine kinases, especially SnRK2 and SnRK3, have activities that link stress and ABA signaling with metabolic signaling (Halford and Hey, 2009; Yu et al., 2014). In Arabidopsis, 38 members of the SnRK family have been described. The different SnRK subfamilies, SnRK1, SnRK2 and SnRK3, have respectively 3, 10 and 25 representatives (Halford and Hey, 2009). All of these SnRKs are associated with metabolic regulations and stress responses. The present transcriptome reveals that *L. perenne* possesses 146 unigenes with high homologies (*e* < 10e-6) with these SnRK subfamilies. In comparison, a recent study has identified 44 SnRKs in *Brachypodium distachyon* that respond to multiple stresses and stress-related signal molecules like ABA, ethylene and H_2_O_2_ (Wang et al., 2015). At least some of *L. perenne* SnRK genes were strongly modulated by low-intensity xenobiotic stress (Table 3, Figure 4, Supplemental Figure 1), thus strengthening the above-discussed link with the involvement of ABA signaling in xenobiotic responses. ABA regulates the activity of SnRK2 and SnRK3 subfamilies which are part of signaling pathways involved in responses to abiotic stresses such as nutrient limitation, drought, cold, salt, and osmotic stresses (Coello et al., 2011). It has also been shown that SnRK2 could be activated by Ca^2+^ (Coello et al., 2012). Among SnRK2-interacting proteins, Protein Phosphatase 2Cs (PP2Cs) act as negative modulators in relation with ABA signaling (Fujita et al., 2013; Danquah et al., 2014). Interestingly, a PP2C-annotated gene was found to be regulated by xenobiotic treatments, at least in the DESeq analysis (Table 3). Moreover, the SnRK2.3-annotated unigene was DE after xenobiotic treatments (Figure 4). DESeq and qRT-PCR analysis also showed that a transcript related to SnRK3.12 (CIPK9) was repressed by G treatment, which, in contrast, induced the expression of a calcium sensing receptor gene and of an aldehyde dehydrogenase gene. Such aldehyde dehydrogenases have been involved in calcium-mediated signaling and stress-regulated detoxification in young plants (Stiti et al., 2011). SnRK3s interact with calcineurin B-like (CBL) calcium-binding proteins, hence their description as CBL-interacting kinases (CIPKs; Coello et al., 2011), and these CIPKs carry out crosstalks between Ca^2+^ and ABA signaling in the responses to abiotic stresses (Yu et al., 2014). Despite some contradictory studies about the sensitivity of plants lacking *CIPK9* on low potassium media, a function in potassium homeostasis has also been suggested (Pandey et al., 2007; Liu et al., 2013b; Yu et al., 2014). All of these data therefore suggest that SnRKs and interacting proteins could be implicated in xenobiotic responses, cell homeostasis under xenobiotic stress and xenobiotic stress adaptation through complex regulations that involve hormone signaling, calcium signaling and potassium homeostasis. Further studies, using hormone inhibitors or antagonists and mutants related to SnRK or hormone signaling, would allow to understand the role of SnRK-related signaling in plant responses to xenobiotics. Interestingly, herbicide resistance in the agricultural weed *Ipomoea purpurea* has been associated with the differential expression of genes involved in such cellular signaling, with high glyphosate treatment inducing an AtPK7-like serine/threonine-protein kinase and repressing genes encoding receptor-like kinases (Leslie and Baucom, 2014).

## Conclusion

The present results are in line with the involvement of genes linked to SnRKs and other protein kinases and to hormonal regulation in response to low-intensity chemical stresses in *L. perenne*. Our results emphasize that responses to xenobiotic stresses require signal transduction mechanisms involving protein-kinases and protein-phosphatases, in accordance with other studies (Sulmon et al., 2007; Fukudome et al., 2014). Our parallel metabolic and molecular approaches establish a vision of responses to mild chemical stress potentially involving complex and interconnected regulation processes. Energy dysfunction is likely to be related to carbohydrate dynamics and regulation. The present results therefore suggested the existence of complex relations between sugar signaling, the signaling pathways associated with phytohormones and calcium (León and Sheen, 2003; Rolland et al., 2006; Kudla et al., 2010; Biswal et al., 2011) and the molecular responses to organic xenobiotics (Ramel et al., 2012; Serra et al., 2013). Finally the close relations between protein-kinase regulations and ABA signaling suggest hormonal actions in response to mild chemical stress. The exact role of these processes in xenobiotic responses remains to be elucidated. This role is likely to be connected to molecular and physiological processes involved in plant growth and development. However, the nature of the primary events leading to such signaling is still completely unknown. Interestingly, herbicide resistance in the agricultural weed *Ipomoea purpurea* has been associated with the differential expression of genes involved in such cellular signaling, with high glyphosate treatment inducing an AtPK7-like serine/threonine-protein kinase and repressing genes encoding receptor-like kinases (Leslie and Baucom, 2014). Situations of NTSR observed in rye-grasses have been correlated with mechanisms of gene expression regulation (Duhoux et al., 2015; Salas et al., 2015). Identification and characterization of key steps of the corresponding signal transduction processes should therefore be of particular interest for herbicide management in crop systems and for restoration-phytoremediation activities in ecological engineering.

### Conflict of interest statement

The authors declare that the research was conducted in the absence of any commercial or financial relationships that could be construed as a potential conflict of interest.

## Abbreviations

G: glyphosate
T: tebuconazole
GT: combination of glyphosate and tebuconazole.
