# Supplementary material for: Genome-Wide Transcriptional Profiling and Metabolic Analysis Uncover Multiple Molecular Responses of the Grass Species Lolium perenne Under Low-Intensity Xenobiotic Stress
Source: Front Plant Sci. 2015 Dec 17;6:1124. doi: 10.3389/fpls.2015.01124 (PMC4681785; doi:10.3389/fpls.2015.01124)
Supplement: Supplementary file 1 [file Table1.PDF]

# *Supplementary Material*

# Large-scale molecular and signaling responses in the grass species *Lolium perenne* under low-intensity xenobiotic stress

**Anne-Antonella Serra[1], Ivan Couée[1], David Heijnen[1], Sophie Coudouel[2], Cécile Sulmon[1], Gwenola Gouesbet[1*]**

[1] *Université de Rennes 1, Centre National de la Recherche Scientifique, UMR 6553 ECOBIO, Campus de Beaulieu, bâtiment 14A, F-35042 Rennes Cedex, France*

[2] *Université de Rennes 1, Centre National de la Recherche Scientifique, UMS 3343 OSUR, Campus de Beaulieu, bâtiment 14A, F-35042 Rennes Cedex, France*

***Correspondence:** Dr Gwenola Gouesbet, UMR 6553 ECOBIO, Campus de Beaulieu, bâtiment 14A, F-35042 Rennes Cedex, France.

gwenola.gouesbet@univ-rennes1.fr

**Supplemental Table 1:**
Selected candidate genes and contigs and corresponding primer sequences for qRT-PCR analysis

| Gene or contig | Forward primer | Reverse primer | Product size | Amplified fragment |
|---|---|---|---|---|
| GAPDH | TCGATGAGGACCTTGTTTCC | GTGCTGTATCCCCACTCGTT | 136 | 901-1036 |
| comp27530_c0_seq1 | GTTCTTTGTTGCCATGTGCT | ATTGCACTCCACAATGCATG | 119 | 169-287 |
| comp2769_c0_seq1 | TCAGGTGACCCATTAGTCCT | CAGCAGTGGAGAGATTGTGT | 84 | 267-350 |
| comp3660_c0_seq1 | GGTGTTGGGTACAGTTGGAT | TAGCCTAGCAGCATTCTTGG | 174 | 733-906 |
| comp4085_c0_seq2 | TGTCAATGGTGGAGAAGGTG | CTACAAACCTTGAGGCCCTT | 126 | 1526-1651 |
| comp5339_c0_seq1 | TGAAGCTTTCCTGGCAGATT | GACAAGCTGGATACCGTCTT | 121 | 402-522 |
| comp6832_c0_seq2 | ACATGGCGAAGATGTAGGTC | CATCTACACCTCCCTGTTCG | 230 | 332-561 |
| comp7152_c0_seq1 | CAACACCATCCTCTTCGACT | CCGTCATCTTGTTCATCAGC | 212 | 382-593 |
| comp7196_c0_seq2 | CACCCTTGCAGGTCTAATGT | AAGACCAGTGCCTGAAATGT | 102 | 243-344 |
| comp7196_c0_seq5 | AAACTGCCCAGAGGTTGTAG | TGTCTGCATGGATGGTGAAA | 80 | 655-734 |
| comp7278_c0_seq1 | CTATGTACGAGGAAGGTGCC | TGGAGAATGCAACATGGACA | 268 | 711-979 |
| comp7296_c0_seq6 | CAAAACCTCCACTGCAGTTG | CTCTTTTCCCTGGCTCAAGT | 245 | 676-920 |
| comp7309_c0_seq3 | CACCCAGATGGAGAGTATGC | TGAATCCAGCCTTTTCAGCT | 196 | 351-546 |
| comp7355_c0_seq2 | TGCATACTGCTTGTGGAACT | ATTGGGATCGATAATGCGCT | 248 | 474-721 |
| comp7388_c0_seq1 | CGAGCAGTCCTTCAACATCT | ATAATCCGCTTTCCTCGACC | 181 | 278-458 |
| comp7427_c0_seq4 | AACTCGATGTAGCCGATCAC | AAGGTGGAGCTGATTGACG | 244 | 336-579 |
| comp7452_c0_seq3 | TAGGAGATTTGGCGACCCTA | AAATGTTCGCTGACTCCTGT | 269 | 30-298 |
| comp7458_c0_seq2 | TCGACAGTGTCTTCAGTTGG | TCAGGAAGGACTGCAAGTTC | 198 | 85-282 |
| comp7489_c0_seq2 | AGCAGTTCTTGAACCCTACG | AAGGGCTCAAACATCGTCAT | 91 | 419-509 |
| comp7493_c0_seq1 | ACCAAGTTCGGTGTAGCAAT | CCATGGTACTCAGCATCTCC | 92 | 238-329 |
| comp7497_c0_seq1 | AGCACAGCAAGGTGTTAGAA | TAGGACCTTCTTCCCATGGT | 227 | 320-546 |
| comp7527_c0_seq1 | TGCCTTCCCTCCTTTAGAGA | CAAATGCCGCTATTGCTGAA | 176 | 782-957 |
| comp7591_c1_seq3_4 | CTCCATCGCGTCCTTCTAC | GTACACGTCCATGTAGTCCC | 118 | 811-928 |
| comp7682_c1_seq3 | TTGCAAGCAGGGTGAAATTG | GACTGACAACACTGTAGGCA | 283 | 491-773 |
| comp7682_c0_seq5 | TGCTTGAGTTCGTGAATGGA | TCCCAAGATGTTGAGGCAAA | 231 | 399-629 |
| comp7697_c0_seq11-14 | GGGCAAGCTCATGATATCGA | AAACTTCTCTCGTGGGTGTC | 109 | 798-906 |
| comp7705_c0_seq3 | CCCAGAGGTTTTACAGGCTT | CGGGACAACTCTTCTTGGAA | 92 | 1047-1138 |
| comp7709_c0_seq1 | GACAGGTTGTCCTTGTTTGC | AAGTGCTGCTATGTGTGGAA | 111 | 1208-1318 |
| comp7722_c0_seq3 | ACAGTTCTTGACGGCATTCT | CATCTCTTCCAGGAAGACCG | 133 | 538-670 |
| comp7188_c0_seq3 | AGCTTTGCCTTGGCCATTAC | ATTGCTTCGTTCAGGTCCAG | 88 | 808-895 |
| comp7528_c0_seq1 | AGCCGGGTATTTAACAGCAT | CTCGCTCGAATTTGTATCGC | 156 | 53-208 |
