# Supplementary material for: Genome-Wide Transcriptional Profiling and Metabolic Analysis Uncover Multiple Molecular Responses of the Grass Species Lolium perenne Under Low-Intensity Xenobiotic Stress
Source: Front Plant Sci. 2015 Dec 17;6:1124. doi: 10.3389/fpls.2015.01124 (PMC4681785; doi:10.3389/fpls.2015.01124)
Supplement: Supplementary file 3 [file Image1.PDF]

Supplementary Material

Large-scale molecular and signaling responses in the grass species *Lolium perenne* under low-intensity xenobiotic stress

Anne-Antonella Serra[1], Ivan Couée[1], David Heijnen[1], Sophie Coudouel[2], Cécile Sulmon[1], Gwenola Gouesbet[1*]

[1] *Université de Rennes 1, Centre National de la Recherche Scientifique, UMR 6553 ECOBIO, Campus de Beaulieu, bâtiment 14A, F-35042 Rennes Cedex, France*

[2] *Université de Rennes 1, Centre National de la Recherche Scientifique, UMS 3343 OSUR, Campus de Beaulieu, bâtiment 14A, F-35042 Rennes Cedex, France*

***Correspondence:** Dr Gwenola Gouesbet, UMR 6553 ECOBIO, Campus de Beaulieu, bâtiment 14A, F-35042 Rennes Cedex, France.

gwenola.gouesbet@univ-rennes1.fr

Supplementary Figure 1.
Multiple sequence alignment of *Lolium perenne*, *Arabidopsis thaliana*, *Oryza sativa* and *Brachypodium distachyon* orthologs

An * (asterisk) indicates positions which have a single, fully conserved residue.
A : (colon) indicates conservation between groups of strongly similar properties - scoring > 0.5 in the Gonnet PAM 250 matrix.
A . (period) indicates conservation between groups of weakly similar properties - scoring =< 0.5 in the Gonnet PAM 250 matrix.
X identical amino acid between *L. perenne* sequences and other sequences

CLUSTAL O(1.2.1) multiple sequence alignment

>prot2769_c0_seq1 *L. perenne*
>CAA19877 protein kinase-like protein Serine/Threonine Kinases, Sucrose nonfermenting 1-related protein kinase subfamily 2, group 3 = STK_SnRK2-3 *A. thaliana*
>Os03g0764800 Serine/threonine-protein kinase SAPK8 *O. sativa*
>XP_003558981.1| PREDICTED: serine/threonine-protein kinase SAPK8-like isoform 1 *B. distachyon*

Serine/threonine-protein kinase = SAPK8 *O. sativa, B. distachyon*
Serine/Threonine Kinases, Sucrose nonfermenting 1-related protein kinase subfamily 2, group 3 = STK_SnRK2-3 *A. thaliana*

```
STKc_SnRK2-3 A. thaliana    ------------MDRPAVSGPMDLPIMHDSDRYELVKDIGSGNFGVARLMRDKQSNELVA 48
SAPK8 O. sativa             MAAAGAGAGAPDRAALTVGPGMDMPIMHDSDRYELVRDIGSGNFGVARLMRDRRTMELVA 60
SAPK8 B. distachyon         ----MAGAAAPDRAALTVGPGMDMPIMHDSDRYELVRDIGSGNFGVARLMRDRGTMELVA 56
prot2769_c0_seq1 L. perenne ----------------------------------------------------RRTMELVA 8
                                                                                : : ****

STKc_SnRK2-3 A. thaliana    VKYIERGEKIDENVKREIINHRSLRHPNIVRFKEVILTPTHLAIVMEYASGGELFERICN 108
SAPK8 O. sativa             VKYIERGEKIDDNVQREIINHRSLKHPNIIRFKEVILTPTHLAIVMEYASGGELFERICK 120
SAPK8 B. distachyon         VKYIERGEKIDENVQREIINHRSLKHPNIIRFKEVILTPTHLAIVMEYASGGELFERICK 116
prot2769_c0_seq1 L. perenne VKYIERGEKIDENVQREIINHRSLKHPNIIRFKEVILTPTHLAIVMEYASGGELFERICK 68
                            ***********:**:*********:****:*****************************:

STKc_SnRK2-3 A. thaliana    AGRFSEDEARFFFQQLISGVSYCHAMQVCHRDLKLENTLLDGSPAPRLKICDFGYSKSSV 168
SAPK8 O. sativa             NVRFSEDEARYFFQQLISGVSYCHSMQVCHRDLKLENTLLDGSPAPRLKICDFGYSKSSV 180
SAPK8 B. distachyon         NVRFGEDEARYFFQQLISGVSYCHSMQVCHRDLKLENTLLDGSPAPRLKICDFGYSKSSV 176
prot2769_c0_seq1 L. perenne NVRFSEDEARYFFQQLISGVSYCHSMQVCHRDLKLENTLLDGSPAPRLKICDFGYSKSSV 128
                              **.*****:*************:***********************************

STKc_SnRK2-3 A. thaliana    LHSQPKSTVGTPAYIAPEVLLKKEYDGKVADVWSCGVTLYVMLVGAYPFEDPEEPKNFRK 228
SAPK8 O. sativa             LHSQPKSTVGTPAYIAPEVLLKKEYDGKTADVWSCGVTLYVMVVGAYPFEDPEEPKNFRK 240
SAPK8 B. distachyon         LHSQPKSTVGTPAYIAPEVLLKKEYDGKIADVWSCGVTLYVMVVGAYPFEDPEEPKNFRK 236
prot2769_c0_seq1 L. perenne LHSQPKSTVGTPAYIAPEVLLKKEYDGKIADVWSCGVTLYVMVVGSYPFEDPEEPKNFRK 188
                            **************************** *************:**:**************

STKc_SnRK2-3 A. thaliana    TIHRILNVQYAIPDYVHISPECRHLISRIFVADPAKRISIPEIRNHEWFLKNLPADLMND 288
SAPK8 O. sativa             TIQRILNVQYSIPENVDISPECRHLISRIFVGDPSLRITIPEIRSHGWFLKNLPADLMDD 300
SAPK8 B. distachyon         TIQRILSVQYSIPDNVDISPECRHLISRIFVGDPALRITIPEIRNHGWFLKNLPADLMDD 296
prot2769_c0_seq1 L. perenne TIQRILNVQYSMPDNVDISPECRHLISRIFVGDPALRITIPEIRNHGWFLKNLPADLIDD 248
                            **:***.***::*: *.**************.**: **:*****.* **********::*

STKc_SnRK2-3 A. thaliana    NTMTTQFDESDQPGQSIEEIMQIIAEATVPPAGTQNLNHYLTDD-DMEEDLE--SDLDDL 345
SAPK8 O. sativa             DSMSSQYEEPDQPMQTMDQIMQILTEATIPPACSR-INHILTDGLDLDDDMDDLDSDSDI 359
SAPK8 B. distachyon         DSMSSQYEEPDQPMQTMDQIMQILTEATIPPACSR-MNRILTGGLDMDDDMDDLESDSDL 355
prot2769_c0_seq1 L. perenne DSMSSQYEEPEQPMQTMDQIMQILTEATIPPACSR-INHILNDGLDMDDDMDDLESDSDL 307
                            ::*::*::* :** *::::****::***:*** :: :*: *.   *:::*::  .. .*:
```

```
STKc_SnRK2-3 A. thaliana    DIDSSGEIVYAM    357
SAPK8 O. sativa             DVDSSGEIVYAM    371
SAPK8 B. distachyon         DIDSSGEIVYAM    367
prot2769_c0_seq1 L. perenne DIDSSGEIVYAM    319
                            *:**********
```

CLUSTAL O(1.2.1) multiple sequence alignment

>prot7355_c0_seq2 *L. perenne*
>NP_171622 CBL-interacting serine/threonine-protein kinase 9 *A. thaliana*
>hypothetical protein CIPK OsI_09813 EEC74432.1 *O. sativa*
>XP_003562157 PREDICTED: CBL-interacting protein kinase 9 isoform X1 *B. distachyon*

CBL-interacting protein kinase 9 = CIPK9

```
CIPK9 A. thaliana           ---------MSGSRRK-ATPASRTRVGNYEMGRTLGEGSFAKVKYAKNTVTGDQAAIKIL 50
CIPK O. sativa              MAEAEAEAAGAGAGAGAGPARRTTRVGRYELGKTIGEGSFAKVKVARDTRTGDTLAIKVL 60
CIPK9 B. distachyon         MAEATAA---AGSEGKRRASSSTRAVGRYELGKTIGEGSFAKVKIARDTRSGAACAIKVL 57
prot7355_c0_seq2 L. perenne ----------------------------YELGKTIGEGSFAKVKIARDTRDGSTCAIKVL 32
                                                        **:*:*:********* *::*  *   ***:*

CIPK9 A. thaliana           DREKVFRHKMVEQLKREISTMKLIKHPNVVEIIEVMASKTKIYIVLELVNGGELFDKIAQ 110
CIPK O. sativa              DRNHVLRHKMVEQIKREISTMKLIKHPNVVQLHEVMASKSKIYMVLEYVDGGELFDKIVN 120
CIPK9 B. distachyon         DRNHVLRHKMVEQIKREIATMKLIKHPNVVQLHEVMASRSKIYMVLEFVDGGELFDKIVN 117
prot7355_c0_seq2 L. perenne DRNHVLRHKMVEQIKREIATMKLIRHPNVVQLHEVMASKTKIYMVLDFVDGGELFDKIVN 92
                            **::*:*******:****:*****:*****:: *****::***:**: *:********.:

CIPK9 A. thaliana           QGRLKEDEARRYFQQLINAVDYCHSRGVYHRDLKPENLILDANGVLKVSDFGLSAFSRQV 170
CIPK O. sativa              SGRLGEDEARRYFHQLINAVDYCHSRGVYHRDLKPENLLLDSHGALKVSDFGLSAFAPQT 180
CIPK9 B. distachyon         SGRLGEDEARRYFHQLINAVDYCHSRGVYHRDLKPENLLLDSYGSLKVSDFGLSAFAPQT 177
prot7355_c0_seq2 L. perenne SGRLGEDEARRYFHQLINAVDYCHSRGVYHRDLKPENLLLDSYGALKVSDFGLSAFAPQT 152
                            .*** ********:************************:**: * ***********: *.

CIPK9 A. thaliana           REDGLLHTACGTPNYVAPEVLSDKGYDGAAADVWSCGVILFVLMAGYLPFDEPNLMTLYK 230
CIPK O. sativa              KEDGLLHTACGTPNYVAPEVLADKGYDGMAADVWSCGIILFVLMAGYLPFDDPNLMTLYK 240
CIPK9 B. distachyon         KEDGLLHTACGTPNYVAPEVLNDKGYDGMAADVWSCGIILFVLMAGYLPFDDPNLMTLYK 237
prot7355_c0_seq2 L. perenne KEDGLLHTACGTPNYVAPEVLADKGYDGMAADVWSCGIILFVLMAGYLPFDDPNLMTLYK 212
                            :******************** ****** ********:*************:********

CIPK9 A. thaliana           RICKAEFSCPPWFSQGAKRVIKRILEPNPITRISIAELLEDEWFKKGYKPPSFDQDDEDI 290
CIPK O. sativa              LICKAKVSCPHWFSSGAKKFIKRILDPNPCTRITIAQILEDDWFKKDYKPPLFEQG-EDV 299
CIPK9 B. distachyon         LIAKANVSCPPWFSSGARNLIKRILDPNPQTRITIAQILEDEWFKKDYKPTDFEQN-EDV 296
prot7355_c0_seq2 L. perenne MITRANVSCPPWLSTGARNLIKRIIDPNPRTRITIAEILEDEWFKKDYKPPQFEQN-EDV 271
                             * :*:.*** *:* **:..****::*** ***:**::***:**** ***  *:*  **:

CIPK9 A. thaliana           TIDDVDAAFSNSKECLVTEKKEKPVSMNAFELISSSSEFSLENLFEKQA-QLVKKETRFT 349
CIPK O. sativa              SLDDVDAAFDCSEENLVAEKREKPESMNAFALISRSQGFNLGNLFEKEMMGMVKRETSFT 359
CIPK9 B. distachyon         SLEDVDAAFNGLEEHLVSEKKEKPESMNAFALISRSQGFNLGNLFEKEMMGMAKRETSFT 356
prot7355_c0_seq2 L. perenne SLEDVDAAFNSSEEHLVAEKREKPESMNAFALISRSQGFNLGNLFEKEMMGLVKRETSFA 331
                            :::******.  :* **:**:*** ***** *** *. *.* *****:   :.*:** *:

CIPK9 A. thaliana           SQRSASEIMSKMEETAKPLGFNVRKDNYKIKMKGDKSGRKGQLSVATEVFEVAPSLHVVE 409
CIPK O. sativa              SQCTPQEIMSKIEEACGPLGFNVRKQNYKMKLKGDKTGRKGHLSVATEVFEVAPSLHMVE 419
CIPK9 B. distachyon         SQRTPQEIMSKIEEACGPLGFNVRKQNYKMKLKGDKTGRKGHLSVATEVFEVAPSLHMVE 416
prot7355_c0_seq2 L. perenne SQRTPQEIVSKIEEACGPLGFNVRKQNYKMKLKGDKTGRKGHLSVATEVFEVAPSLHMVE 391
                            ** : .**:**:**:. ********:***:*:****:****:***************:**

CIPK9 A. thaliana           LRKTGGDTLEFHKFYKNFSSGLKDVVWNTDAAAEEQKQ-       447
CIPK O. sativa              LRKTGGDTLEFHNFYNNFSSELKDIVWKSESDAKAAKKR       458
CIPK9 B. distachyon         LRKTGGDTLEFHNFYQSFSSELKDIVWKSESDTRR----       451
prot7355_c0_seq2 L. perenne LRKTGGDTLEFHSFYKSFSTELKDIVWKSESDTITKQKR       430
                            ************.**:.**: ***:**:::: :
```

>prot7296_c0_ seq6 *L. perenne*
>NP_567574 STKc_MAK_like Kinase-like Serine/Threonine Kinases *A. thaliana*
>NP_001056617 STKc_MAK_like Os06g0116100 *O. sativa*
>XP_010228082 STKc_MAK_like cyclin-dependent kinase F-4-like *B. distachyon*

Kinase-like Serine/Threonine Kinases = STKc_MAK_like

```
STKc_MAK_like A. thaliana    MDRYKLIKEVGDGTFGSVWRAINKQTGEVVAIKKMKKKYYSWDECINLREVKSLRRMNHP 60
STKc_MAK_like O. sativa      MERYKIIKEVGDGTFGSVWRAINKESGEVVAIKKMKKKYYSWEECINLREVKSLRRMNHP 60
STKc_MAK_like B. distachyon  MERYNVITEVGDGTFGSVWRAINKENGEVVAIKKMKKKYYSWDECINLREVKSLRKMNHP 60
prot7296_c0_seq6 L. perenne MERYNIITEVGDGTFGSVWRAINKESGEVVAIKKMKKKYYSWEECINLREVKSLRKMNHP 60
                             *:**::*.****************:.****************:************:****

STKc_MAK_like A. thaliana    NIVKLKEVIRENDILYFVFEYMECNLYQLMKDRQKLFAEADIKNWCFQVFQGLSYMHQRG 120
STKc_MAK_like O. sativa      NIVKLKEVIRENDMLFFVFEYMECNLYQLMKSRGKPFSETEVRNWCFQIFQALSHMHQRG 120
STKc_MAK_like B. distachyon  NIVKLKEVIREHDMLFFVFEYMECNLYQLMKNKGKPFSETEIRNWCFQIFQALSHMHQRG 120
prot7296_c0_seq6 L. perenne NIVKLKEVIRENDMLFFVFEYMECNLYQLMKSKGKPFSETEIRNWCFQVFQALSHMHQRG 120
                             ***********.*:*:***************.: *  *:*::::*****:**.**:*****

STKc_MAK_like A. thaliana    YFHRDLKPENLLVSKDIIKIADFGLAREVNSSPPFTEYVSTRWYRAPEVLLQSYVYTSKV 180
STKc_MAK_like O. sativa      YFHRDLKPENLLVTKELIKIADFGLAREISSEPPYTEYVSTRWYRAPEVLLQASVYNSAV 180
STKc_MAK_like B. distachyon  YFHRDLKPENLLVTKEVIKVADFGLAREISSEPPYTEYVSTRWYRAPEVLLQASVYSSAV 180
prot7296_c0_seq6 L. perenne YFHRDLKPENLLVTKELIKVADFGLAREIISEPPYTEYVSTRWYRAPEVLLQASFYSSAV 180
                             *************:*::**:********: *.**:*****************: .*.* *

STKc_MAK_like A. thaliana    DMWAMGAIMAELLSLRPIFPGASEADEIYKICSVIGTPTEETWLEGLNLANTINYQFPQL 240
STKc_MAK_like O. sativa      DMWAMGAIIAELFSLRPLFPGSNEADEIYKICSILGTPNQRTWAEGLQLAASIRFQFPQS 240
STKc_MAK_like B. distachyon  DMWAMGAIIAELFSLRPLFPGSSEADEIYKICSILGTPNPRTWAEGLQLAASINFQFPQL 240
prot7296_c0_seq6 L. perenne DMWAMGAIIAELFSLRPLFPGSSEADEIYKICSILGTPNQHTWAKGLQLAASINFQFPQS 240
                             ********:***:****:***:.**********::***.  .**  :**:**  :*.:****

STKc_MAK_like A. thaliana    PGVPLSSLMPSASEDAINLIERLCSWDPSSRPTAAEVLQHPFFQSCFYVPPSLRPKP--S 298
STKc_MAK_like O. sativa      GSIHLSEVVPSASEDAISLISWLCSWDPQRRPTAVEVLQHPFFQPCFYIPPSLRFRSTNG 300
STKc_MAK_like B. distachyon  ESIHLSEVVPSASEDAVNLISWLCSWDPRRRPTAVEVLQHPFFQPCFYIPPSLRFRST-G 299
prot7296_c0_seq6 L. perenne ESIQLSEMVPSASEDAVNLISWLCSWDPCKRPTAVEVLQHPFFQPCFYIPPSLRFRST-G 299
                              .: **.::*******:.**. ******  ****.********* ***:***** :   .

STKc_MAK_like A. thaliana    VARTPPPVGPRGSFEHQSVKRYPVSLA-NAKP--FNSYVSPKSNAAFGSGVQRKLDMVNQ 355
STKc_MAK_like O. sativa      YAATPPSVGAKGAVDQKNARRYSVGPLSNGRPAVNYSYLS-ANTPARAAGVQRKLELDHQ 359
STKc_MAK_like B. distachyon  YATTPPSAGARGALDLKNTRRYPAGTLSNEKPTVNYSYMSTTNTPARAAGVQRKLELDHQ 359
prot7296_c0_seq6 L. perenne YPATPPSVGAKGALDQKNARRYPVGTLSNGRPAVNNSYLS-TNTPARAAGVQRKLELDHQ 358
                                *** .* :*:.: :..:** ..    * :*    **:*   .. * .:******:: .*

STKc_MAK_like A. thaliana    DGTRNTKPVRSS---VRDSKYRPPGKKS-PPAASLNK--NRVTRSS---VSETADKLANM 406
STKc_MAK_like O. sativa      VNMNSCQAPEGNHKLTKAEAMNQPWSRPPPAAAAVRSNGNYFTKDQGPRAPDIAEKLSQL 419
STKc_MAK_like B. distachyon  VKV------ESNHKLMKENAMNQPWSRPPPP--AVRSNGNYLTKDQSPRAPDIAEKLSQL 411
prot7296_c0_seq6 L. perenne MKL------ESNHKLTKENAMNQPWSRLPPP---VRSNGNYLAKDQITHAPDLAEKLSQL 409
                                       ...    : .  . * .:  *    :..  * .::..   . : *:**:::

STKc_MAK_like A. thaliana    TIGATGSRRHSV--------SVVGQH-QQLKPPPMKAGWVGETRDMFLRPTQ-PTTNA-Y 455
STKc_MAK_like O. sativa      TVGSNRVPSLASDKFVDMKARTHGNTMKRPLPPVGTRTWHAPA-DPFRRPYEMPGDRAFL 478
STKc_MAK_like B. distachyon  TMASNRTPVLSSDRFADMKARAHGDTARRPLP-LGSRAWRAPT-DPFRRTYEMPGERALL 469
prot7296_c0_seq6 L. perenne SMASNRAPVLSSDKFADLKGRTHGDAVRQPLP-LGSRAWHAPN-DPFRRTYEMPGERALL 467
                             ::.:.     :              . *:  ::  *    .  * .    * * *  : *  .*

STKc_MAK_like A. thaliana    SRKVAG   461
STKc_MAK_like O. sativa      PRKLVS   484
STKc_MAK_like B. distachyon  QRKLVS   475
prot7296_c0_seq6 L. perenne QRKLVS   473
                              **:..
```

>prot6832_c0_seq2 *L. perenne*

>NP_191283 beta-1,3-glucanase 3 *A. thaliana*

>XP_010232797 PREDICTED: glucan endo-1,3-beta-glucosidase GIII-like isoform X2 *B. distachyon*

>AAL35900 endo-1,3-beta-glucanase *O. sativa*

putative beta-1,3-endoglucanase = β-1,3-EG

```
β-1,3-EG A. thaliana         MKMCNGSSFLASLPLLLLLLSFILASFFDTAVGQIGVCYGRNGNNLRPASEVVALYQQRN 60
β-1,3-EG B. distachyon       --MAKQASVASVLGAALVLI-VALLAAFPAAVHSIGVCNGVLGSNLPTPSDVVQLYKSKG 57
β-1,3-EG O. sativa           --MARRQGVASMLTI--ALI-IGAFASAPTTVQSIGVCYGVLGNNLPSRSEVVQLYKSKG 55
prot6832_c0_seq2 L. perenne -------------NF--DQL-VH--VRSSTPAMVAGVCYGMVANNLPSRSDVVQMYRSKG 42
                                                 :  .        :  .   *** *   ..**    *:** :*:.:
```

```
β-1,3-EG A. thaliana        IRRMRLYDPNQETLNALRGSNIELVLDVPN-PDLQRLASSQAEADTWVRNNVRNYA---N 116
β-1,3-EG B. distachyon      IASMRIYAPETGILRALAGTGIGLVMDVPN-ENLTAMASSPPFAAAWVKANVQPYSSSSG 116
β-1,3-EG O. sativa          INGMRIYYPDKEALNALRNSGIALILDVG--DQLSYLAASSSNAAAWVRDNVKPYYP--A 111
prot6832_c0_seq2 L. perenne ITGMRLYSPDAQALSALRNSGISLMIDVGGTDQLAYLAASASNASAWVRTNVQAY-Q--G 99
                            *  **:* *:    * **   : * *::**     :*  :*:*    * :**: **: *

β-1,3-EG A. thaliana        VTFRYISVGNEVQPSDQAASFVLPAMQNIERAVSSLG---IKVSTAIDTRGI-SGFPPSS 172
β-1,3-EG B. distachyon      VSFRYIAVGNEVVDSEGQ-KNILPAMKNLAGALAASGIG-IKVSTALRFDAITNTFPPSN 174
β-1,3-EG O. sativa          VNIKYIAVGNEVEGG-AT-NSILPAIRNVNSALASSGLGAIKASTAVKFDVISNSYPPSA 169
prot6832_c0_seq2 L. perenne LTIKYIVAGNEVQGG-DT-QNIVPAIRNLNAALSAVGLGGIKVSTSVRFDVVANSYPPSA 157
                            :.::** .****   .     . ::**::*:   *::: *    **.**::     :  . :***

β-1,3-EG A. thaliana        GTFTPEFRSFIAPVISFLSSKQSPLLVNNYPYFSYTGNMRDIRLDYTLFTAPSTVVNDGQ 232
β-1,3-EG B. distachyon      GVFSD--PSFMGPVAAYLASTGAPLLVNVYPYFAYVDNPRDIQLGYATFQPGTTVRDDGN 232
β-1,3-EG O. sativa          GVFRD---AYMKDIARYLATTGAPLLANVYPYFAYRGNPRDISLNYATFRPGTTVRDPNN 226
prot6832_c0_seq2 L. perenne GVFAQ---AYMTDVARLLSSTGAPLLANVYPYFAYRDNPRDIQLNYATFRPGTTVRDDNN 214
                            *.*      :::  :    *::. :***.* ****:*   * *** * *: *    :** :  :

β-1,3-EG A. thaliana        -NQYRNLFHAILDTVYASLEKAGGGSLEIVVSESGWPTAGGAATGVDNARTYVNNLIQTV 291
β-1,3-EG B. distachyon      GLVYTNLFDAMVDSIYAALEDAGTPGVGVVVSESGWPSAGGFAATAENARRYNQGLIGHV 292
β-1,3-EG O. sativa          GLTYTNLFDTMVDAVYAALEKAGAGNVKVVVSESGWPSAGGFGASVDNARAYNQGLIDHV 286
prot6832_c0_seq2 L. perenne GLIYTSLFDAMVDAIYAALEKAGTPGLRVVISESGWPSASGFAATADNARAYNQGLIDHV 274
                               * .**.:::*::**:**.**    : :*:******:*.* .: .:*** * : **   *

β-1,3-EG A. thaliana        KNGSPRRPGRATETYIFAMFDENSKQGPETEKFWGLFLPNLQPKYVVNFN   341
β-1,3-EG B. distachyon      GGGTPKKAGP-LETYVFAMFNENQKTGLETEKHFGLFNPDKSPAYSISF-   340
β-1,3-EG O. sativa          GRGTPKRPGP-LEAYIFAMFNENQKNGDPTERNFGLFYPNKSPVYPIRF-   334
prot6832_c0_seq2 L. perenne GGGTPKRPGT-LETYIFAMFNENFKLGDLVEKHFGLFNPDKSPAYPIRF-   322
                              *:*:: *    *:*:****:** * *   .*: :*** *: .* * : *
```

>prot7709_c0_seq1 *L. perenne*

>NP_195027 glycine dehydrogenase (decarboxylating) 2 *A. thaliana*

>BAD35509 putative glycine dehydrogenase *O. sativa*

>XP_003569669 PREDICTED: glycine dehydrogenase (decarboxylating) 2, mitochondrial-like *B. distachyon*

glycine dehydrogenase (decarboxylating) = GLDP

```
GLDP 2 A. thaliana          ------------------------------MERARRLAYRGIVKRLVNDTKRHRNAETPH 30
GLDP O. sativa              ------------------------------MERARRLANRALLRRLLAAATAES------ 24
GLDP 2 B. distachyon        ------------------------------MERARRLANRALLRRLLAATTSTT------ 24
prot7709_c0_seq1 L. perenne STPTSALLVRSGGRLLLPPPLPASLPNPNPMERARRLANRALLRRLLAASSSTT------ 54
                                                          ******** *.:::**:  :.

GLDP 2 A. thaliana          LVPHAPARYVSSLSPFISTPRSVNHTAAFGRHQQTRSISVDAVKPSDTFPRRHNSATPDE 90
GLDP O. sativa              --PAAPSRGISTLAKG-SRPRAPPRPAPHQYTTGRRPVSASALQPSDTFPRRHNSATPAE 81
GLDP 2 B. distachyon        --SPSPSRGISTLVPA-PAAGSRPHTRRAHQHTQGRPVSVSALQPSDTFPRRHNSATPAE 81
prot7709_c0_seq1 L. perenne --SPSPSRGISTLVPK-PAPAGPRPRRAHHQHTPSRPVSVSALQPSDTFPRRHNSATPAE 111
                                :*:* :*:*        .              * :*..*::************* *

GLDP 2 A. thaliana          QTHMAKFCGFDHIDSLIDATVPKSIRLDSMKFS-KFDAGLTESQMIQHMVDLASKNKVFK 149
GLDP O. sativa              QAAMASECGFGTVDALIDATVPAAIRAPEMRFSGRFDAGFTESEMIEHMQRLAAMNRAYK 141
GLDP 2 B. distachyon        QAVMASACGFNTLDALIDATVPAAIRAPPMQFSGKFDAGFTESQMLEHMARLSSMNKAYK 141
prot7709_c0_seq1 L. perenne QAVMASACGFNTLDSLIDATVPAAIRAPPMQFTGKFDAGFTESQMLDHMAHLASMNKVYK 171
                            *: **. ***  :*:******* :**   *:*: :****:***:*::**  *:: *:.:*

GLDP 2 A. thaliana          SFIGMGYYNTHVPTVILRNIMENPAWYTQYTPYQAEISQGRLESLLNFQTVITDLTGLPM 209
GLDP O. sativa              SFIGMGYYNTHVPAVILRNLMENPAWYTQYTPYQAEIAQGRLESLLNYQTMVADLTGLPM 201
GLDP 2 B. distachyon        SFIGMGYYNTHIPAVILRNLMENPAWYTQYTPYQAEIAQGRLESLLNYQTMVADLTGLPM 201
prot7709_c0_seq1 L. perenne SFIGMGYYNTHIPAVILRNLMENPAWYTQYTPYQAEIAQGRLESLLNYQTMVADLTGLPM 231
                            ***********:*:*****:*****************:*********:**:::*******

GLDP 2 A. thaliana          SNASLLDEGTAAAEAMAMCNNILKGKKKTFVIASNCHPQTIDVCKTRADGFDLKVVTSDL 269
GLDP O. sativa              SNASLLDEATAAAEAMAMCNGILKSKKKTFLIASNCHPQTIDVCQTRAAGFDLNVVVADA 261
GLDP 2 B. distachyon        SNASLLDEATAAAEAMAMCNGILKSKKKTFLIASNCHPQTIDICQTRAAGFDLNVVVSAA 261
prot7709_c0_seq1 L. perenne SNASLLDEATAAAEAMAMCNGILKSKKKTFLIASNCHPQTIDICKTRASGFDLNVVVSDA 291
                            ********.*********** ***.*****:***********:*:*** ****:**.:

GLDP 2 A. thaliana          KDIDYSSGDVCGVLVQYPGTEGEVLDYAEFVKNAHANGVKVVMATDLLALTVLKPPGEFG 329
GLDP O. sativa              KDFDYGSGDVCGVLVQYPGTEGEVLDYAEFVRDAHAHGVKVVMATDLLALTSLRPPGEIG 321
GLDP 2 B. distachyon        KDFDYSSGDVCGVLVQYPGTEGEVLDYAEFVKDAHAHGVKVVMATDLLALTTLRPPGEIG 321
prot7709_c0_seq1 L. perenne KDFDYSSGDVCGVLVQYPGTEGEVLDYAEFVKDAHKHGVKVVMATDLLALTTLRPPGEIG 351
                            **:**.*************************::** .************* *:****:*
```

```
GLDP 2 A. thaliana          ADIVVGSAQRFGVPMGYGGPHAAFLATSQEYKRMMPGRIIGISVDSSGKQALRMAMQTRE 389
GLDP O. sativa              ADIAVGSAQRFGVPMGYGGPHAAFLATSQEYKRLMPGRIIGVSVDSSGKPALRMAMQTRE 381
GLDP 2 B. distachyon        ADIAVGSAQRFGVPMGYGGPHAAFLATSQEYKRLMPGRIIGVSVDSSGKPALRMAMQTRE 381
prot7709_c0_seq1 L. perenne ADIAVGSAQRFGVPMGYGGPHAAFLATSQEYKRLMPGRIIGVSVDSSGKPALRMAMQTRE 411
                            ***.*****************************:*******:******* **********

GLDP 2 A. thaliana          QHIRRDKATSNICTAQALLANMAAMYAVYHGPAGLKSIAQRVHGLAGIFSLGLNKLGVAE 449
GLDP O. sativa              QHIRRDKATSNICTAQALLANMAAMYAVYHGPEGLKAIADRVHGLAGTFAQGLKKLGTVT 441
GLDP 2 B. distachyon        QHIRRDKATSNICTAQALLANMAAMYAVYHGPAGLKAIADRVHGLAGTFAHGLKKLGTVT 441
prot7709_c0_seq1 L. perenne QHIRRDKATSNICTAQALLANMAAMYAVYHGPAGLKAIADRVHGLAGTFAHGLKKLGTVT 471
                            ******************************** ***:**:******* *: **:***..

GLDP 2 A. thaliana          VQELPFFDTVKIKCSDAHAIADAASKSEINLRVVDSTTITASFDETTTLDDVDKLFKVFA 509
GLDP O. sativa              VQELPFFDTVKVKVADANAIAQEACKNEMNLRVVDATTITVAFDETTTLEDVDKLFKVFN 501
GLDP 2 B. distachyon        VQELPYFDTVKVTCADANAIAEEARKNEMNLRVVDANTITVAFDETTTLEDVDKLFKVFS 501
prot7709_c0_seq1 L. perenne VQELPYFDTVKITCADANAIAEEARKNEMNLRVVDANTITVAFDETTTLEDVDKLFKVFS 531
                            *****:*****:.  :**.***: * *.*:******:.***.:*******:*********

GLDP 2 A. thaliana          SGKPVPFTAESLAPEVQNSIPSSLTRESPYLTHPIFNMYHTEHELLRYIHKLQSKDLSLC 569
GLDP O. sativa              GGKPVNFTAESLAPEVSSSIPSSLVRKSPYLTHPIFNMYHTEHELLRYLYKLQSKDLSLC 561
GLDP 2 B. distachyon        GGKPVDFTAESIAPEVSSSIPSSLVRNSPYLTHPIFSMYHTEHELLRYLHKLQSKDLSLC 561
prot7709_c0_seq1 L. perenne GGKPVGFTAESIAPEVSSSIPSSLVRDSPYLTHPIFSMYHTEHELLRYLHKLQNKDLSLC 591
                            .**** *****:****..******.*.*********.***********::***.******

GLDP 2 A. thaliana          HSMIPLGSCTMKLNATTEMMPVTWPSFTDIHPFAPVEQAQGYQEMFENLGDLLCTITGFD 629
GLDP O. sativa              HSMIPLGSCTMKLNATVEMMPVTYPNFANMHPFAPTDQAAGYHEMFDDLGDLLCKITGFD 621
GLDP 2 B. distachyon        HSMIPLGSCTMKLNATVEMMPVTDPNFANMHPFAPIDQAAGYHEMFDNLGELLNTITGFD 621
prot7709_c0_seq1 L. perenne HSMIPLGSCTMKLNATVEMMPVTDPKFANMHPFAPTDQAAGYHEMFDNLGDLLNTITGFD 651
                            ****************.****** *.*:::*****  :**  **:***::**:**  .*****

GLDP 2 A. thaliana          SFSLQPNAGAAGEYAGLMVIRAYHMSRGDHHRNVCIIPVSAHGTNPASAAMCGMKIITVG 689
GLDP O. sativa              SFSLQPNAGASGEYAGLMVIRAYHRARGDYHRDVCIIPVSAHGTNPASAAMCGMKIVAVG 681
GLDP 2 B. distachyon        SFSLQPNAGASGEYAGLMVIRAYHRSRGDHHRNVCIIPVSAHGTNPASAAMCGMKIVAVG 681
prot7709_c0_seq1 L. perenne SFSLQPNAGASGEYAGLMVIRAYHRARGDHHRNVCIIPVSAHGTNPASAAMCGMKIITVG 711
                            **********:************* :***:**:***********************::**

GLDP 2 A. thaliana          TDAKGNINIEEVRKAAEANKDNLAALMVTYPSTHGVYEEGIDEICNIIHENGGQVYMDGA 749
GLDP O. sativa              TDSKGNINIEELRKAAEANKDNLAALMVTYPSTHGVYEEGIDEICMIIHENGGQVYMDGA 741
GLDP 2 B. distachyon        TDSKGNINIEELRKAAEANKDNLSALMVTYPSTHGVYEEGIDEICRIIHENGGQVYMDGA 741
prot7709_c0_seq1 L. perenne TDSKGNINIPELKKAAEANKDNLSALMVTYPSTHGVYEEGIDEICRIIHENGGQVYMDGA 771
                            **:****** *::**********:********************* **************

GLDP 2 A. thaliana          NMNAQVGLTSPGFIGADVCHLNLHKTFCIPHGGGGPGMGPIGVKNHLAPFLPSHPVIPTG 809
GLDP O. sativa              NMNAQVGLTSPGFIGADVCHLNLHKTFCIPHGGGGPGMGPIGVKKHLAPFLPSHPVITTG 801
GLDP 2 B. distachyon        NMNAQVGLTSPGFIGADVCHLNLHKTFCIPHGGGGPGMGPIGVKKHLAPFLPSHPVIPTG 801
prot7709_c0_seq1 L. perenne NMNAQVGLTSPGFIGADVCHLNLHKTFCIPHGGGGPGMGPIGVKKHLAPFLPSHPVIPTG 831
                            ********************************************:************ **

GLDP 2 A. thaliana          GIPQPEKTAPLGAISAAPWGSALILPISYTYIAMMGSGGLTDASKIAILNANYMAKRLEK 869
GLDP O. sativa              GFPLPEKTDPLGTISAAPWGSALILPISYTYIAMMGSKGLTDASKIAILNANYMTKRLEK 861
GLDP 2 B. distachyon        GFPLPEKTDPLGSISAAPWGSALILPISYTYIAMMGSQGLTEASKIAILNANYMAKRLEK 861
prot7709_c0_seq1 L. perenne GFPLPEKTDPLGSISAAPWGSALILPISYTYIAMMGSQGLTDASKIAILNANYMAKRLEK 891
                            *:* **** ***:************************ ***:************:*****

GLDP 2 A. thaliana          HYPVLFRGVNGTVAHEFIIDLRGFKNTAGIEPEDVAKRLMDYGFHGPTMSWPVPGTLMIE 929
GLDP O. sativa              HYPVLFRGVNGTVAHEFIIDLRGFKTTAGIEPEDVAKRLMDYGFHAPTMSWPVPGTLMIE 921
GLDP 2 B. distachyon        HYPVLFRGVNGTVAHEFIIDLRGFKATAGIEPEDVAKRLMDYGFHGPTMSWPVPGTLMIE 921
prot7709_c0_seq1 L. perenne HYPVLFRGVNGTVAHEFIIDLRGFKATAGIEPEDVAKRLMDYGFHGPTMSWPVPGTLMIE 951
                            ************************* *******************.**************

GLDP 2 A. thaliana          PTESESKAELDRFCDALISIREEIAQIEKGNADVQNNVLKGAPHPPSLLMADTWKKPYSR 989
GLDP O. sativa              PTESESKAELDRFCDALISIREEIAEIESGKADVNNNVLKSAPHPPQLLMSDSWTKPYSR 981
GLDP 2 B. distachyon        PTESESKAELDRFCDALISIREEIAEVENGKADAHNNVLKGAPHPPQLLMSDAWTKPYSR 981
prot7709_c0_seq1 L. perenne PTESESKAELDRFCDALISIREEIAQVENGVADANNNVLKGAPHPPQLLMSDAWTKPYSR 1011
                            *************************::*.* **..*****.*****.***:*:*.*****

GLDP 2 A. thaliana          EYAAFPAPWLRSSKFWPTTGRVDNVYGDRKLVCTLLPEEEQVAAAVSA---- 1037
GLDP O. sativa              EYAAFPAAWLRGAKFWPTTCRVDNVYGDRNLICTLQQGSQVAEEAAAATA-- 1031
GLDP 2 B. distachyon        EYAAFPAAWLRGAKFWPTTCRVDNVYGDRNLICTLQQASQVTEEAAAAATA 1033
prot7709_c0_seq1 L. perenne EYAAFPAAWLRGAKFWPTTCRVDNVYGDRNLICTLQQASQVAEEAAAATA-- 1061
                            ******* ***.:****** *********:*:***   .: .  *.:*
```

>prot7722_c0_seq5 *L. perenne*
>NP_181241phenylalanine ammonia-lyase 1 *A. thaliana*
>PAL2_ORYSI Phenylalanine ammonia-lyase *O. sativa*
>XP_003580144 PREDICTED: phenylalanine ammonia-lyase *B. distachyon*

Phenylalanine ammonia-lyase = PAL

```
PAL A. thaliana             MEINGAHKSNGGGVDAMLCGGDIKTKNMVINAEDPLNWGAAAEQMKGSHLDEVKRMVAEF 60
PAL O. sativa               MECENGRVSAN-GMS------GLCVA---APRADPLNWGKATEEMTGSHLDEVKRMVAEY 50
PAL B. distachyon           MEYENGHAATY-G-D------GLCVAAPLAPRADPLNWGKAAEELSGSHLDAVKRMVEEY 52
prot7722_c0_seq5 L. perenne ------------------------------------------------------------ 0

PAL A. thaliana             RKPVVNLGGETLTIGQVAAISTIGNSVKVELSETARAGVNASSDWVMESMNKGTDSYGVT 120
PAL O. sativa               RQPLVKIEGASLRIAQVAAVAA-AGEARVELDESARERVKASSDWVMNSMMNGTDSYGVT 109
PAL B. distachyon           RRPVVKMEGASLTIAQVAAVAA-GAEARVELDESARGRVKESSDWVMNSMMNGTDSYGVT 111
prot7722_c0_seq5 L. perenne ------------------------------------------------------------ 0

PAL A. thaliana             TGFGATSHRRTKNGVALQKELIRFLNAGIFGSTKETSHTLPHSATRAAMLVRINTLLQGF 180
PAL O. sativa               TGFGATSHRRTKEGGALQRELIRFLNAGAFGTG-TDGHVLPAEATRAAMLVRINTLLQGY 168
PAL B. distachyon           TGFGATSHRRTKEGGALQRELIRFLNAGAFGTG-EDGHVLPAAATRAAMLVRVNTLLQGY 170
prot7722_c0_seq5 L. perenne ------------------------------------------------------------ 0

PAL A. thaliana             SGIRFEILEAITSFLNNNITPSLPLRGTITASGDLVPLSYIAGLLTGRPNSKATGPNGEA 240
PAL O. sativa               SGIRFEILEAIAKLLNANVTPCLPLRGTITASGDLVPLSYIAGLVTGRENAVAVAPDGSK 228
PAL B. distachyon           SGIRFEILETIATLLNANVTPCLPLRGTITASGDLVPLSYIAGLVTGRPNSVATAPDGRK 230
prot7722_c0_seq5 L. perenne ------------------------------------------------------------ 0

PAL A. thaliana             LTAEEAFKLAGISSGFFDLQPKEGLALVNGTAVGSGMASMVLFETNVLSVLAEILSAVFA 300
PAL O. sativa               VNAAEAFKIAGIQGGFFELQPKEGLAMVNGTAVGSGLASTVLFEANILAILAEVLSAVFC 288
PAL B. distachyon           VNAAEAFKIAGIQHGFFELQPKEGLAMVNGTAVGSGLASMVLFEANILGVLAEVLSAVFC 290
prot7722_c0_seq5 L. perenne ----EAFKIAGIQHGFFELQPKEGLAMVNGTAVGSGLASMVLFEANILSLLAEVLSAVFC 56
                                ****:***. ***:********:*********:** ****:*:*.:***:*****.

PAL A. thaliana             EVMSGKPEFTDHLTHRLKHHPGQIEAAAIMEHILDGSSYMKLAQKLHEMDPLQKPKQDRY 360
PAL O. sativa               EVMNGKPEYTDHLTHKLKHHPGQIEAAAIMEHILEGSSYMKHAKKLGELDPLMKPKQDRY 348
PAL B. distachyon           EVMNGKPEFTDHLTHKLKHHPGQIEAAAIMEHILEGSSYMMLAKKLGELDPLMKPKQDRY 350
prot7722_c0_seq5 L. perenne EVMNGKPEFTDHLTHKLKHHPGQIEAAAIMEHILEGSSYMKLAKKLGELDPLMKPKQDRY 116
                            ***.****:******:******************:*****  *:** *:*** *******

PAL A. thaliana             ALRTSPQWLGPQIEVIRYATKSIEREINSVNDNPLIDVSRNKAIHGGNFQGTPIGVSMDN 420
PAL O. sativa               ALRTSPQWLGPQIEVIRAATKSIEREINSVNDNPLIDVSRGKALHGGNFQGTPIGVSMDN 408
PAL B. distachyon           ALRTSPQWLGPQIEVIRAATKSIEREINSVNDNPLIDVSRGKAIHGGNFQGTPIGVSMDN 410
prot7722_c0_seq5 L. perenne ALRTSPQWLGPQIEVIRAATKSIEREINSVNDNPLIDVSRGKAIHGGNFQGTPIGVSMDN 176
                            ***************** ********************** **:****************

PAL A. thaliana             TRLAIAAIGKLMFAQFSELVNDFYNNGLPSNLTASRNPSLDYGFKGAEIAMASYCSELQY 480
PAL O. sativa               TRLAIAAIGKLMFAQFSELVNDFYNNGLPSNLSGGRNPSLDYGFKGAEIAMASYCSELQF 468
PAL B. distachyon           TRLAIAAIGKLMFAQFSELVNDFYNNGLPSNLSGGRNPSLDYGFKGAEIAMASYCSELQF 470
prot7722_c0_seq5 L. perenne TRLAIAAIGKLMFAQFSELVNDFYNNGLPSNLSGGRNPSLDYGFKGAEIAMASYCSELQF 236
                            ********************************:..************************:

PAL A. thaliana             LANPVTSHVQSAEQHNQDVNSLGLISSRKTSEAVDILKLMSTTFLVAICQAVDLRHLEEN 540
PAL O. sativa               LGNPVTNHVQSAEQHNQDVNSLGLISSRKTAEAIDILKLMSSTFLIALCQAVDLRHIEEN 528
PAL B. distachyon           LGNPVTNHVQSAEQHNQDVNSLGLISSRKTAEAIDILKLMSSTFLVALCQAIDLRHLEEN 530
prot7722_c0_seq5 L. perenne LGNPVTNHVQSAEQHNQDVNSLGLISSRKTAEAIDILKLMSSTFLVALCQAIDLRHIEEN 296
                            *.****.***********************:**:*******:***:*:***:****:***

PAL A. thaliana             LRQTVKNTVSQVAKKVLTTGVNGELHPSRFCEKDLLKVVDREQVYTYADDPCSATYPLIQ 600
PAL O. sativa               VKSAVKSCVMTVAKKTLSTNSTGDLHVARFCEKDLLKEIDREAVFAYADDPCSHNYPLMK 588
PAL B. distachyon           VRSAVKNCVTTVARKTLSTNVNGHLHNARFCEKDLLLTIDREAVFAYADDPCSANYPLMQ 590
prot7722_c0_seq5 L. perenne VKNAVKNCVKTVARKTLSTNDSGHLHSARFCEKDLLLTIDREAVFAYADDPCSANYPLMQ 356
                            ::.:**. *  **:*.*:*  .*.** :********  :*** *::******* .***::

PAL A. thaliana             KLRQVIVDHALINGESEKNAVTSIFHKIGAFEEELKAVLPKEVEAARAAYDNGTSAIPNR 660
PAL O. sativa               KLRNVLVERALANGAAEFNADTSVFAKVAQFEEELRATLPGAIEAARAAVENGTAAIPSR 648
PAL B. distachyon           KMRAVLVEHALANGEAERDVETSVFAKLAAFEQELRAVLPKEVEAARAAVENGTATKQNR 650
prot7722_c0_seq5 L. perenne KMRAVLVEHALANGEAERDAQTSVFAKLATFEQELRAVLPKEVEAARCSVENGTAAQQNR 416
                            *:* *:*::** ** :* :. **:* *:. **:**:*.**  :****.: :***::  .*
```

```
PAL A. thaliana             IKECRSYPLYRFVREELGTELLTGEKVTSPGEEFDKVFTAICEGKIIDPMMECLNEWNGA 720
PAL O. sativa               ITECRSYPLYRFVREELGTKYLTGEKTRSPGEELNKVLVAINEGKHIDPLLECLKEWNGE 708
PAL B. distachyon           IAECRSYPLYRFVREELGTEYLTGEKTRSPGEEVDKVFVAMNQGKHIDALLECLKEWNGE 710
prot7722_c0_seq5 L. perenne ITECRSYPLYRFVRKELGTEYLTGEKTRSPGEEVDKVFVAMNQGKHINALLECLK----- 471
                            * ************:****: *****. *****.:**:.*: :** *: ::***:

PAL A. thaliana             PIPIC    725
PAL O. sativa               PLPIC    713
PAL B. distachyon           PLPLC    715
prot7722_c0_seq5 L. perenne -----    471
```

>prot7196_c0_seq2 *L. perenne*

>NP_001190823 NADPH--cytochrome P450 reductase 1 *A. thaliana*

>BAD05443 putative cytochrome P450 reductase *O. sativa*

>XP_003579401 PREDICTED: NADPH--cytochrome P450 reductase-like *B. distachyon*

NADPH--cytochrome P450 reductase 1 = P450 reductase 1

```
P450 reductase1 A. thaliana  ----MTSALYASDLFKQLKSIMGTDSLSDDVVLVIATTSLALVAGFVV-LLWKKTTADRS 55
P450 reductase1 O. sativa    ------------------------------------------------------------ 0
P450 reductase1B. distachyon MALEAARSWAASVLPPELAAAA-----GGDPLVALAATAAALVAGLLVLAVWFRSGGGAP 55
prot7196_c0_seq2 L. perenne  ------------------------------------------------------------ 0


P450 reductase1 A. thaliana  GELKPLMIPKSLMAKDEDDDLDLGSGKTRVSIFFGTQTGTAEGFAKALSEEIKARYEKAA 115
P450 reductase1 O. sativa    ------------------------------------------------------------ 0
P450 reductase1B. distachyon --SKPAATPLRPPPVKVDADADADDGRKRVTIFFGTQTGTAEGFAKAMAEEAKVRYEKTV 113
prot7196_c0_seq2 L. perenne  ------------------------------------------------------------ 0


P450 reductase1 A. thaliana  V----KDDYAADDDQYEEKLKKETLAFFCVATYGDGEPTDNAARFYKWFTEENERDIKLQ 171
P450 reductase1 O. sativa    ----------MEDEEYEERLKKEKISLFFVATYGDGEPTDNAARFYKWFTEGNERGVWLN 50
P450 reductase1B. distachyon FKVVDLDDYAAEDDEYEEKLKKETLVLFFLATYGDGEPTDNAARFYKWFTEGKEKEVWLK 173
prot7196_c0_seq2 L. perenne  ------------------------------------------------------------ 0


P450 reductase1 A. thaliana  QLAYGVFALGNRQYEHFNKIGIVLDEELCKKGAKRLIEVGLGDDDQSIEDDFNAWKESLW 231
P450 reductase1 O. sativa    DFQYAIFGLGNRQYEHFNKVAKVVDELLVEQGGKRLVPVGLGDDDQCIEDDFNAWKETLW 110
P450 reductase1B. distachyon DFKYAVFGLGNRQYEHFNKVAKVVDELLEEQGGKRLVPCGLGDDDQCIEDDFTAWKEQVW 233
prot7196_c0_seq2 L. perenne  ------------------------------------SPVCLGDDDQCIEDDFTAWKELVW 24
                                                                     ******.*****.**** :*

P450 reductase1 A. thaliana  SELDKLLKDEDDKS-VATPYTAVIPEYRVVTHDPRFTTQKSMESNVANGNTTIDIHHPCR 290
P450 reductase1 O. sativa    PELDQLLRDENDVS-TGTTYTAAIPEYRVEFVKPDEAAHLERNFSLANGYAVHDAQHPCR 169
P450 reductase1B. distachyon PELDQLLRDDDDTTGASTPYTAAIPEYRVVFIDKSDLVVEDKSWTLANGNGVIDIHHPCR 293
prot7196_c0_seq2 L. perenne  PELDQLLRDDDDTTGASTPYTAAIPEYRIVFIDKSDLVFEDKSWTLANGNGVIDAQHPCR 84
                              ***:**:*::* : ..* ***.*****:    .     .  . . .:***   . * :****

P450 reductase1 A. thaliana  VDVAVQKELHTHESDRSCIHLEFDISRTGITYETGDHVGVYAENHVEIVEEAGKLLGHSL 350
P450 reductase1 O. sativa    ANVAVRRELHTPASDRSCTHLEFDIAGTGLTYETGDHVGVYTENCLEVVEEAERLLGYSP 229
P450 reductase1B. distachyon SNVAVRKELHKPASDRSCIHLEFDISGTGLVYETGDHVGVYSENSVDTVEQAERLLGLSP 353
prot7196_c0_seq2 L. perenne  SNVALRKELHKPASDRSCIHLEFDISGTGLVYETGDHVGVYAENSIETVEQAETLLGLTP 144
                              :**:::***.  ***** ******: **:.**********:** :: **:*  *** :

P450 reductase1 A. thaliana  DLVFSIHADKEDGSPLES-AVPPPFPGPCTLGTGLARYADLLNPPRKSALVALAAYATEP 409
P450 reductase1 O. sativa    EAFFTIHADKEDGTPLGGGSLAPPFPSPITVRNALARYADLLNSPKKSALVALATYASDS 289
P450 reductase1B. distachyon DTVFSIHADAEDGSPRKGGSLAPPFPSPCTLRTALLRYADLLNSPKKAALVALASHASDP 413
prot7196_c0_seq2 L. perenne  DTVFSIHADAEDGSPRKGGSLAPPFPSPCTLRTALLRYADLLSSPKKAALTALAAHASDL 204
                             : .*:**** ***:*  . :: ****.* *: ..* ******. *:*:**.***::*::

P450 reductase1 A. thaliana  SEAEKLKHLTSPDGKDEYSQWIVASQRSLLEVMAAFPSAKPPLGVFFAAIAPRLQPRYYS 469
P450 reductase1 O. sativa    TEADRLRFLASPAGKDEYAQWVVASQRSLLEVMAEFPSAKPPLGVFFAAVAPRLQPRYYS 349
P450 reductase1B. distachyon TEAERLRFLASPAGKDEYSQWIVASQRSLLEVMAAFPSAKPPLGVFFAAVAPRLQPRYYS 473
prot7196_c0_seq2 L. perenne  TEAERLRFLASPAGKDEYAQWIVASQRSLIEVMAAFPSAKPPLGVFFAAVAPRLQPRFYS 264
                             :**::*:.*:** *****:**:*******:**** *************:*******:**

P450 reductase1 A. thaliana  ISSSPRLAPSRVHVTSALVYGPTPTGRIHKGVCSTWMKNAVPAEKSHECSGAPIFIRASN 529
P450 reductase1 O. sativa    ISSSPSMAPTRIHVTCALVHEKTPAGRVHKGVCSTWIKNAIPSEETKDCSWAPVFVRQSN 409
P450 reductase1B. distachyon ISSSPKMAPSRIHVTCALVYGPTPTERIHQGVCSTWMKNTLPLEYSEECSWAPIFVRQSN 533
prot7196_c0_seq2 L. perenne  ISSSPKMAPSRIHVTCALVYGPTPTE---------------------------------- 290
                             ***** :**:*:***.***:  **:
```

```
P450 reductase1 A. thaliana FKLPSNPSTPIVMVGPGTGLAPFRGFLQERMALKEDGEELGSSLLFFGCRNRQMDFIYED 589
P450 reductase1 O. sativa   FKLPADPSVPVIMIGPGTGLAPFRGFLQERLSQKQSGAELGRSVFFFGCRNSKMDFIYED 469
P450 reductase1B. distachyon FKLPADPSTPVIMIGPGTGLAPFRGFLQERLALKESGVGLGTSILFFGCRNRNMDYIYED 593
prot7196_c0_seq2 L. perenne ------------------------------------------------------------ 290


P450 reductase1 A. thaliana ELNNFVDQGVISELIMAFSREGAQKEYVQHKMMEKAAQVWDLIKEEGYLYVCGDAKGMAR 649
P450 reductase1 O. sativa   ELNTFLEEGALSELVLAFSREGPTKEYVQHKMSQKASEIWDMISQGGYIYVCGDAKGMAR 529
P450 reductase1B. distachyon ELQNFLQEGALSELVVAYSREGPTKEYVQHKMVEKATEIWNIISQGGYVYVCGDAKGMAR 653
prot7196_c0_seq2 L. perenne ------------------------------------------------------------ 290


P450 reductase1 A. thaliana DVHRTLHTIVQEQEGVSSSEAEAIVKKLQTEGRYLRDVW       688
P450 reductase1 O. sativa   DVHRVLHTIVQEQGSLDSSKAESFVKSLQTEGRYLRDVW       568
P450 reductase1B. distachyon DVHRVLHTIVQEQGSLDNSKTESYVKSLQMEGRYLRDVW       692
prot7196_c0_seq2 L. perenne ---------------------------------------       290
```

>prot7278_c0_seq1 *L. perenne*

>NP_196867 magnesium chelatase subunit H *A. thaliana*

>CHLH_ORYSI Full=Magnesium-chelatase subunit ChlH *O. sativa*

>XP_010234592 PREDICTED: magnesium-chelatase subunit ChlH, chloroplastic *B. distachyon*

Magnesium-chelatase subunit ChlH = Mg-chelatase H

```
Mg-chelatase H A. thaliana  -MASLVYSPFTLSTSKAEHLSSLTNSTKHSFLRKKHRS----TKPAKSFFKVKSAVSGNG 55
Mg-chelatase H O. sativa    -MSSLVSTPFTTATG-VQKKLGAP-VPLHSFLLSRRQPAAGAGRGRAAAAAIRCAVAGNG 57
Mg-chelatase H B. distachyonMSSSLVSAPFAPAATSRAQRKRLS-APLHSFLLSRRSHNQQQHASGTRPGTIRCAVAGNG 59
prot7278_c0_seq1 L. perenne ------------------------------------------------------------ 0


Mg-chelatase H A. thaliana  LFTQTNPEVRRIVPIKR----DNVPTVKIVYVVLEAQYQSSLSEAVQSLNKTS-RFASYE 110
Mg-chelatase H O. sativa    LFTQTKPEVRRVVPPEGDASRRGVPRVKVVYVVLEAQYQSSVTAAVRELNADPRRAAGFE 117
Mg-chelatase H B. distachyonLFTQTNPDVRRVVPPRGL-----LPRVKIVYVVLEAQYQSSVTAAVQSLNADPRRAAEFE 114
prot7278_c0_seq1 L. perenne ------------------------------------------------------RAAEFE 6
                                                                                  * * :*

Mg-chelatase H A. thaliana  VVGYLVEELRDKNTYNNFCEDLKDANIFIGSLIFVEELAIKVKDAVEKERDRMDAVLVFP 170
Mg-chelatase H O. sativa    VVGYLVEELRDEETYKTFCADLADANVFIGSLIFVEELALKVKDAVEKERDRMDAVLVFP 177
Mg-chelatase H B. distachyonVSGYLVEELRDASTYESFKEDLSDANVFIGSLIFVEELAVKVRDAVERERDRMDAVLVFP 174
prot7278_c0_seq1 L. perenne VVGYLVEELRDVDTYAAFCDDVAAANVFIGSLIFVEEAALKVRDAVAQHRDRMDAVLVFP 66
                            * ********* .**  *  *:  **:********** *:**:*** :.***********

Mg-chelatase H A. thaliana  SMPEVMRLNKLGSFSMSQLGQSKSPFFQLFKRKKQGSAGFADSMLKLVRTLPKVLKYLPS 230
Mg-chelatase H O. sativa    SMPEVMRLNKLGSFSMSQLGQSKSPFFQLFKRK-KNSGGFADSMLKLVRTLPKVLKYLPS 236
Mg-chelatase H B. distachyonSMPEVMRLNKLGSFSMSQLGQSKSPFFQLFKRNKKNSAGFADSMLKLVRTLPKVLKYLPS 234
prot7278_c0_seq1 L. perenne SMPEVMRLNKLGSFSMAQLGQSKSPFFQLFKRNKKDSSGFADSMLKLVRTLPKVLKYLPS 126
                            ****************:***************: : *.**********************

Mg-chelatase H A. thaliana  DKAQDARLYILSLQFWLGGSPDNLQNFVKMISGSYVPALKGV-KIEYSDPVLFLDTGIWH 289
Mg-chelatase H O. sativa    DKAQDARLYILSLQFWLGGSPDNLQNFLKMIAVSYVPALKGA-DIKYDDPVLFLDAGIWH 295
Mg-chelatase H B. distachyonDKAQDARLYILSLQFWLGGSPDNLQNFLKMIAVSYVPALKGNPGITYDDPVLFLDAGIWH 294
prot7278_c0_seq1 L. perenne DKAQDARLYILSLQFWLGGSPDNLQNFLKMIAVSYVPALKGA-DIKYDDPVLFLDTGIWH 185
                            ***************************:***: ********   * *.*******:****

Mg-chelatase H A. thaliana  PLAPTMYDDVKEYWNWYDTRRDTNDSLKRKDATVVGLVLQRSHIVTGDDSHYVAVIMELE 349
Mg-chelatase H O. sativa    PLAPTMYDDVKEYLNWYGTRRDTNDKLKDPNAPVIGLVLQRSHIVTGDDGHYVAVIMELE 355
Mg-chelatase H B. distachyonPLAPTMYDDVKEYLNWYGTRRDADERLKDPDAPIIGLVLQRSHIVTGDDGHYVAVIMELE 354
prot7278_c0_seq1 L. perenne PLAPTMYDDVKEYLNWYDTRRDTTGKLKDPDAPVIGLVLQRSHIVTGDDGHYVAVIMELE 245
                            ************* *** ****:   **  :* ::**************.**********

Mg-chelatase H A. thaliana  ARGAKVVPIFAGGLDFSGPVEKYFVDPVSKQPIVNSAVSLTGFALVGGPARQDHPRAIEA 409
Mg-chelatase H O. sativa    AKGAKVIPIFAGGLDFSGPTQRYLVDPITGKPFVNAVVSLTGFALVGGPARQDHPKAIAA 415
Mg-chelatase H B. distachyonARGAKVIPIFAGGLDFSGPTEKYLVDSVTKKPFVDAVVSLTGFALVGGPARQDHPKAIAA 414
prot7278_c0_seq1 L. perenne ARGAKVIPIFAGGLDFSGPTQQYLVDSITKKPFVNAVVSLTGFALVGGPARQDHPKAIAA 305
                            *:****:************.::*:** :: :*:*::.******************:** *

Mg-chelatase H A. thaliana  LKKLDVPYLVAVPLVFQTTEEWLNSTLGLHPIQVALQVALPELDGAMEPIVFAGRDPRTG 469
Mg-chelatase H O. sativa    LQKLDVPYIVALPLVFQTTEEWLNSTLGLHPIQVALQVALPELDGGMEPIVFAGRDPRTG 475
Mg-chelatase H B. distachyonLQKLDVPYIVALPLVFQTTEEWLNSTLGLHPIQVALQVALPELDGGMEPIVFAGRDPRSG 474
prot7278_c0_seq1 L. perenne LQKLDVPYIVALPLVFQTTEEWLNSTLGLHPIQVALQVALPELDGGMEPIVFSGRDPRSG 365
                            *:******:**:*********************************.******:*****:*
```

```
Mg-chelatase H A. thaliana  KSHALHKRVEQLCIRAIRWGELKRKTKAEKKLAITVFSFPPDKGNVGTAAYLNVFASIFS 529
Mg-chelatase H O. sativa    KSHALHKRVEQLCTRAIRWAELKRKTKEEKKLAITVFSFPPDKGNVGTAAYLNVFNSIYS 535
Mg-chelatase H B. distachyonKSHALHKRVEQLCTRAVRWAQLKRKTKEDKRLAITVFSFPPDKGNVGTAAYLNVFSSIYS 534
prot7278_c0_seq1 L. perenne KSHALHKRVEQLCTRAIRWAVLKRKTKDAKKLAITVFSFPPDKGNVGTAAYLNVFSSIYS 425
                            ************* **:**. ******  *:************************ **:*

Mg-chelatase H A. thaliana  VLRDLKRDGYNVEGLPENAETLIEEIIHDKEAQFSSPNLNVAYKMGVREYQDLTPYANAL 589
Mg-chelatase H O. sativa    VLQDLKKDGYNVEGLPDTAEALIEEVIHDKEAQFNSPNLNVAYRMNVREYQSLTSYASLL 595
Mg-chelatase H B. distachyonVLRDLKKDGYNVDGLPETPEELIEEVIHDKEAQFNSPNLNVVYRMNVREYQALTPYASML 594
prot7278_c0_seq1 L. perenne VLKDLKKDGYNVDGLPDTPEELIEEVIHDKEAQFSSPNLNVVYRMNVREYQALTPYASML 485
                            **:***:*****:***:. * ****:********.******.*:* ***** ** **. *

Mg-chelatase H A. thaliana  EENWGKPPGNLNSDGENLLVYGKAYGNVFIGVQPTFGYEGDPMRLLFSKSASPHHGFAAY 649
Mg-chelatase H O. sativa    EENWGKPPGNLNSDGENLLVYGKQYGNVFIGVQPTFGYEGDPMRLLFSKSASPHHGFAAY 655
Mg-chelatase H B. distachyonEENWGKAPGHLNSDGENLLVYGKQYGNIFIGVQPTFGYEGDPMRLLFSKSASPHHGFAAY 654
prot7278_c0_seq1 L. perenne EENWGKAPGHLNSDGENLLVYGKQYGNIFIGVQPTFGYEGDPMRLLFSKSASPHHGFAAY 545
                            ****** **.************* ***:********************************

Mg-chelatase H A. thaliana  YSYVEKIFKADAVLHFGTHGSLEFMPGKQVGMSDACFPDSLIGNIPNVYYYAANNPSEAT 709
Mg-chelatase H O. sativa    YTFVEKIFQADAVLHFGTHGSLEFMPGKQVGMSDACYPDSLIGNIPNIYYYAANNPSEAT 715
Mg-chelatase H B. distachyonYTYVEKIFKADAVLHFGTHGSLEFMPGKQVGMSDACYPDSLIGNIPNIYYYAANNPSEAT 714
prot7278_c0_seq1 L. perenne YTYVEKIFKADAVLHFGTHGSLEFMPGKQVGMSDACYPDSLIGNIPNIYYYAANNPSEAT 605
                            *::*****:***************************:**********:************

Mg-chelatase H A. thaliana  IAKRRSYANTISYLTPPAENAGLYKGLKQLSELISSYQSLKDTGRGPQIVSSIISTAKQC 769
Mg-chelatase H O. sativa    VAKRRSYANTISYLTPPAENAGLYKGLKQLSELISSYQSLKDTGRGPQIVSSIISTAKQC 775
Mg-chelatase H B. distachyonVAKRRSYANTISYLTPPAENAGLYKGLKQLSELIASYQSLKDTGRGNQIVSSIISTARQC 774
prot7278_c0_seq1 L. perenne VAKRRSYANTISYLTPPAENAGLYKGLKQLSELIASYQSLKDTGRGNQIVSSIISTAKQC 665
                            :*********************************:*********** **********:**

Mg-chelatase H A. thaliana  NLDKDVDLPDEGLELSPKDRDSVVGKVYSKIMEIESRLLPCGLHVIGEPPSAMEAVATLV 829
Mg-chelatase H O. sativa    NLDKDVPLPEEGVELPPNERDLIVGKVYAKIMEIESRLLPCGLHVIGEPPSAIEAVATLV 835
Mg-chelatase H B. distachyonNLDKDVDLPEEGEELPANERDLVVGKVYAKIMEIESRLLPCGLHVIGEPPTAVEAVATLV 834
prot7278_c0_seq1 L. perenne NLDKDVPLPDEGVELPPNERDLVVGKVYGKLMEIESRLLPCGLHVIGEPPTAVEAVATLV 725
                            ****** **:** **  ::** :*****.*:*******************:*:*******

Mg-chelatase H A. thaliana  NIAALDRPEDEISALPSILAECVGREIEDVYRGSDKGILSDVELLKEITDASRGAVSAFV 889
Mg-chelatase H O. sativa    NIASLDRPEDEIYSLPNILAQTVGRNIEDVYRGSDKGILADVELLRQITEASRGAITAFV 895
Mg-chelatase H B. distachyonNIAALDRPEENIYALPGILAATVGRTIEDVYRGSDKGVLDDVELLKQITEASRGAVGAFV 894
prot7278_c0_seq1 L. perenne NIAALDRPEENIYALPGILAATVGRTIEDVYRGSDKGVLADVELLKQITEASRGAVGAFV 785
                            ***:*****::* :** ***  *** ***********:* *****::**:*****: ***

Mg-chelatase H A. thaliana  EKTTNSKGQVVDVSDKLTSLLGFGINEPWVEYLSNTKFYRANRDKLRTVFGFLGECLKLV 949
Mg-chelatase H O. sativa    ERTTNNKGQVVDVTNKLSTMLGFGLSEPWVQHLSKTKFIRADREKLRTLFTFLGECLKLI 955
Mg-chelatase H B. distachyonEKSTNSKGQVVDVSNKLSSILGFSLSEPWVEYLSQTKFIRADREKLRNLFGFLGECLKLI 954
prot7278_c0_seq1 L. perenne EKTTNSKGQVVDVKNKLSSILGFGLSEPWVEYLSTTKFIRADREKLRNCFAFLGECLKLI 845
                            *::**.*******.:**:::***.:.****::** *** **:*:***. * ********:

Mg-chelatase H A. thaliana  VMDNELGSLMQALEGKYVEPGPGGDPIRNPKVLPTGKNIHALDPQAIPTTAAMASAKIVV 1009
Mg-chelatase H O. sativa    VADNELGSLKLALEGSYVEPGPGGDPIRNPKVLPTGKNIHALDPQAIPTTAALKSAKIVV 1015
Mg-chelatase H B. distachyonVADNELGALKLALDGSYVEPGPGGDPIRNPKVLPTGKNIHALDPQSIPTAAAMKSAKIVV 1014
prot7278_c0_seq1 L. perenne VADNELGALKLALEGSYVEPGPGGDPIRNPKVLPTGKNIHALDPQSIPTAAAMKSAKVVV 905
                            * *****:*  **:*.*****************************:***:**: ***:**

Mg-chelatase H A. thaliana  ERLVERQKLENEGKYPETIALVLWGTDNIKTYGESLGQVLWMIGVRPIADTFGRVNRVEP 1069
Mg-chelatase H O. sativa    DRLLERQKVDNGGKYPETIALVLWGTDNIKTYGESLAQVLWMIGVRPVADTFGRVNRVEP 1075
Mg-chelatase H B. distachyonDRLLERQKADNGGKYPETIALVLWGTDNIKTYGESLAQVMWMVGVLPVSDAFGRVNRVEP 1074
prot7278_c0_seq1 L. perenne ERLLERQKADNGGKYPETIALVLWGTDNIKTYGESLAQVFWMLGVEPVTDGLGRVNRVEP 965
                            :**:**** :* ************************.**:**:** *::* :********

Mg-chelatase H A. thaliana  VSLEELGRPRIDVVVNCSGVFRDLFINQMNLLDRAIKMVAELDEPVEQNFVRKHALEQAE 1129
Mg-chelatase H O. sativa    VSLEELGRPRIDVVVNCSGVFRDLFINQMNLLDRAVKMVAELDEPEEMNYVRKHAQEQAR 1135
Mg-chelatase H B. distachyonVSLEELGRPRIDVVVNCSGVFRDLFINQMNLLDRAVKLVAELDEPAEMNFVRKHALEQAA 1134
prot7278_c0_seq1 L. perenne VSIEELGRPRVDVVVNCSGVFRDLFINQMNLLDRAVKMVAELDEPVEMNYVRKHALEQAE 1025
                            **:*******:************************:*:******* * *:***** ***

Mg-chelatase H A. thaliana  ALGIDIREAATRVFSNASGSYSANISLAVENSSWNDEKQLQDMYLSRKSFAFDSDAPGAG 1189
Mg-chelatase H O. sativa    ELGVSLREAATRVFSNASGSYSSNVNLAVENASWTDEKQLQDMYLSRKSFAFDCDAPGAG 1195
Mg-chelatase H B. distachyonELGVSVREAATRVFSNASGSYSSNVNLAVENATWTDEKQLQDMYLSRKSFAFDSDAPGVG 1194
prot7278_c0_seq1 L. perenne ELGVSVREAATRVFSNASGSYSSNVNLAVENATWTDEKQLQDMYLSRKSFAFDSDAPGLG 1085
                             **:.:****************:*:.*****::*.******************.**** *

Mg-chelatase H A. thaliana  MAEKKQVFEMALSTAEVTFQNLDSSEISLTDVSHYFDSDPTNLVQSLRKDKKKPSSYIAD 1249
Mg-chelatase H O. sativa    MREQRKTFELALATADATFQNLDSSEISLTDVSHYFDSDPTKLVQGLRKDGRAPSSYIAD 1255
Mg-chelatase H B. distachyonMLEKRKTFELALATADATFQNLDSSEISLTDVSHYFDSDPTKLVQGLRKDGRAPASYIAD 1254
prot7278_c0_seq1 L. perenne MLEKRKTFELALATAEATFQNLDSSEISLTDVSHYFDSDPTKLVQGLRKDGRAPSSYIAD 1145
                            * *:::.**:**:**:.************************:***.**** : *:*****
```

```
Mg-chelatase H A. thaliana  TTTANAQVRTLSETVRLDARTKLLNPKWYEGMMSSGYEGVREIEKRLSNTVGWSATSGQV 1309
Mg-chelatase H O. sativa    TTTANAQVRTLSETVRLDARTKLLNPKWYEGMMKSGYEGVREIEKRLTNTVGWSATSGQV 1315
Mg-chelatase H B. distachyonTTTANAQVRTLSETMRLDARTKLLNPRWYEGMMKSGYEGVREIEKRLTNTVGWSATSGQV 1314
prot7278_c0_seq1 L. perenne TTTANAQVRTLSETMRLDARTKLLNPRWYEGMMKSGYEGVREIEKRLTNTVGWSATSGQV 1205
                            **************:***********:******.*************:************

Mg-chelatase H A. thaliana  DNWVYEEANSTFIQDEEMLNRLMNTNPNSFRKMLQTFLEANGRGYWDTSAENIEKLKELY 1369
Mg-chelatase H O. sativa    DNWVYEEANATFIEDEAMRKRLMDTNPNSFRKLVQTFLEASGRGYWETSEENLEKLRELY 1375
Mg-chelatase H B. distachyonDNWVYEEANTTFIEDEEMRKRLMDTNPNSFRKLLQTFLEANGRGYWDTSEDNLERLRELY 1374
prot7278_c0_seq1 L. perenne DNWVYEEANTTFIEDEEMRKRLMDTNPNSFRKLLQTFLEANGRGYWETSEDNLERLRELY 1265
                            *********:***:** * :***:********::******.*****:** :*:*:*:***

Mg-chelatase H A. thaliana  SQVEDKIEGIDR     1381
Mg-chelatase H O. sativa    SEVEDKIEGIDR     1387
Mg-chelatase H B. distachyonSEVEDKIEGIDR     1386
prot7278_c0_seq1 L. perenne SEVEDKIEGIDR     1277
                            *:**********
```

>prot7497_c0_seq2 *L. perenne*

>OSJNBB0060M15.2 hypothetical protein ATP-sulfurylase *O. sativa*

>XP_003580616 PREDICTED ATP sulfurylase 2-like *B. distachyon*

>AAM63309 ATP sulfurylase 2 *A. thaliana*

ATP sulfurylase 2-like = ATPS2-like

```
ATPS-like O. sativa         ------------------------------------------------------------ 0
ATPS2-like B. distachyon    MAVHLLTA-----PRLHSSSSSPAPLPRRRATASAPLAHPLLLLHSRLRLATTAAVSRPV 55
ATPS2 A. thaliana           MSLMIRSSYVSHITLFQPRNSKPSSFTNQISFLSSSNNNPFLNLVY----------KRNL 50
prot7497_c0_seq2 L. perenne ------------------------------------------------------------ 0


ATPS-like O. sativa         ------------------------------------------------------------ 0
ATPS2-like B. distachyon    PRARRAMSAIRSSLIDPDGGALVDLVAPADRRASLRAEAEALPRVGLAPIDVEWAHVLAE 115
ATPS2 A. thaliana           TMQSVSKMTVKSSLIDPDGGELVELIVPETEIGVKKAESETMPKVKLNQIDLEWVHVISE 110
prot7497_c0_seq2 L. perenne ------------------------------------------------------------ 0


ATPS-like O. sativa         ---------MREHEYLQSLHFNCIRLPDGAGVVNMSLPIVLAIGDREKEEIGSSPDVALH 51
ATPS2-like B. distachyon    GWASPLRGFMREHEYLQCIHFNSLRLPAG-GVVNMSLPIVLAIGDREKDNIGDTPDVALA 174
ATPS2 A. thaliana           GWASPLKGFMREDEYLQSLHFNSLRLKNG-TFVNMSLPIVLAIDDDTKEQIGSSENVALV 169
prot7497_c0_seq2 L. perenne ------------------------------------------------------------ 0


ATPS-like O. sativa         GPDGAVLAILRRVEIYPHNKEERIARTWGTTAPGLPYVDEAIAQAGNWLIGGDLEVIEPI 111
ATPS2-like B. distachyon    GPDGQLLAILRRVEIYPHNKEERIARTWGTTAPGLPYVDEAITPAGNWLIGGDLEVLEPI 234
ATPS2 A. thaliana           CPQGDIIGSLRSVEIYKHNKEERIARTWGTTSPGLPYVEEYITPSGNWLIGGDLEVFEPI 229
prot7497_c0_seq2 L. perenne -----LLAVLRSVEIYPHNKEERIARTWGTTAPGLPYVDEAITSAGNWLIGGDLEVLQPI 55
                                 ::. ** **** **************:******:* *: :***********::**

ATPS-like O. sativa         KYNDGLDHYRLSPQQLRNEFDKRGADAVFAFQLRNPVHNGHALLMNDTRRRLLEMGFKNP 171
ATPS2-like B. distachyon    KYNDGLDHYRLSPQQLRDEFDKRGADAVFAFQLRNPVHNGHALLMNDTRRRLLEMGFKNP 294
ATPS2 A. thaliana           KYNDGLDHYRLSPKQLREEFDNRQADAVFAFQLRNPVHNGHALLMNDTRKRLLEMGYKNP 289
prot7497_c0_seq2 L. perenne KYNDGLDHYRLSPQQLRDEFDKRGADAVFAFQLRNPVHNGHALLMNDTRRRLLEMGFKNP 115
                            *************:***:***:* *************************:******:***

ATPS-like O. sativa         ILLLHPLGGFTKADDVPLPVRMEQHSKVLEDGVLDPETTIVSIFPSPMHYAGPTEVQWHA 231
ATPS2-like B. distachyon    ILLLHPLGGFTKADDVPLPVRMEQHSKVLEDGVLDPETTIVSIFPSPMHYAGPTEVQWHA 354
ATPS2 A. thaliana           VLLLHPLGGFTKADDVPLDVRMEQHSKVLEDGVLDPKTTIVSIFPSPMHYAGPTEVQWHA 349
prot7497_c0_seq2 L. perenne ILLLHPLGGFTKADDVPLPVRMEQHSKVLEDGVLDPETTVVSIFPSPMHYAGPTEVQWHA 175
                            :***************** *****************:**:********************

ATPS-like O. sativa         KARINAGANFYIVGRDPAGMGHPTEKRDLYNPDHGKKVLSMAPGLEKLNILPFKVAAYDT 291
ATPS2-like B. distachyon    KARINAGANFYIVGRDPAGMGHPTEKRDLYNPDHGKKVLSMAPGLEKLNILPFKVAAYDT 414
ATPS2 A. thaliana           KARINAGANFYIVGRDPAGMGHPTEKRDLYDPDHGKRVLSMAPGLEKLNILPFRVAAYDT 409
prot7497_c0_seq2 L. perenne KARINAGANFYIVGRDPAGMGHPTEKRDLYNPDHGKKVLSMAPGLEKLNILPFKVAAYDT 235
                            ******************************:*****:****************:******

ATPS-like O. sativa         VAKKMAFFDPSRSKDFLFISGTKMRAFAKSGENPPDGFMCPGGWKVLVDYYNSLQTEEAA 351
ATPS2-like B. distachyon    VAKKMAFFEPSRSQDFLFISGTKMRTFAKTGENPPDGFMCPGGWKVLVDYYNSLQTEEAA 474
ATPS2 A. thaliana           IEKKMAFFDPSRAKEFLFISGTKMRTYARTGENPPDGFMCPSGWNVLVKYYESLQEKEAK 469
prot7497_c0_seq2 L. perenne VAKKMAFFEPSRSQDFLFISGTKMRTFAKTGENPPDGFMCPSGWKVLVDYYNSLQTEEAA 295
                            : ******:***:::**********::*::***********.**:***.**:*** :**
```

```
ATPS-like O. sativa         VATV-------       355
ATPS2-like B. distachyon    IAAAIV---         480
ATPS2 A. thaliana           QQAVVSA--         476
prot7497_c0_seq2 L. perenne ATAAAPATV         338
                              :.
```

>prot7527_c0_seq1 *L. perenne*

>NP_194903 putative protein phosphatase 2C 59 *A. thaliana*

>Os02g0149800 hypothetical protein Serine/threonine phosphatases, family 2C *O. sativa*

>XP_003574690 probable protein phosphatase 2C 10 *B. distachyon*

protein phosphatase 2C = PP2C

```
putative PP2C A. thaliana   -------------------------------------------------------MGYLN 5
putative PP2C O. sativa     MHGRPSR-PLASSSSSSSSRVFSFFLAPRVFLFLVVVVVVVFLPGRSSCWW---LEGT-- 54
probable PP2C B. distachyon MHERSAGIPPTLAAAASAAA---------RFSFLLL--LLLVVAGRSSSAWSWCREGEAE 49
prot7527_c0_seq1 L. perenne ------------------------------------------------------------ 0


putative PP2C A. thaliana   --SVLSSSSQVHSDDGPVSGGGLSQNGKFSYGYASSPGKRSSMEDFYETRIDGVEGEIVG 63
putative PP2C O. sativa     --EELEEEMGFAGDCSPVSGGGLSENGKFSYGYASAPGKRASMEDFYETRIDGVDGETIG 112
probable PP2C B. distachyon EVKGCSRIMGFGGDGSPATGGGISENGRFSYGFASCAGKRASMEDFYETRVDDVDGETVG 109
prot7527_c0_seq1 L. perenne ----------FRSDGSPATGGGISEKGRFSYGFASCPGKRASMEDFYETRVDDVDGETVG 50
                                      . .* .*.:***:*::*:****:**. ***:*********:* *:** :*

putative PP2C A. thaliana   LFGVFDGHGGARAAEYVKQNLFSNLIRHPKFISDTTAAIADAYNQTDSEFLKSENSQNRD 123
putative PP2C O. sativa     LFGVFDGHGGARAAEYVKQHLFSNLIKHPKFISDIKSAIAETYNHTDSEFLKAESSHTRD 172
probable PP2C B. distachyon LFGVFDGHGGARAAEYVKKHLFSNLIKHPQFIADTKSAIAETFTHTDSEFLKADSSHTRD 169
prot7527_c0_seq1 L. perenne LFGVFDGHGGARAAEYVKKHLFSNLIKHPKFMTETNAAIAETFNHTDSEFLKADSSHSRD 110
                            ******************:.******:**:*::: .:***:::.:*******::.*:.**

putative PP2C A. thaliana   AGSTASTAILVGDRLLVANVGDSRAVICRGGNAIAVSRDHKPDQSDERQRIEDAGGFVMW 183
putative PP2C O. sativa     AGSTASTAILVGDRLLVANVGDSRAVVCRGGDAIAVSRDHKPDQSDERQRIEDAGGFVMW 232
probable PP2C B. distachyon AGSTASTAILVGGRLVVANVGDSRAVVCKGGKAIAVSRDHKPDQTDERQRIEEAGGFVMW 229
prot7527_c0_seq1 L. perenne AGSTASTAILVGGRLVVANVGDSRAVVSKGGKAIAVSRDHKPDQTDERQRIEEAGGFVMW 170
                            ************ **:**********:.:**.************:*******:*******


putative PP2C A. thaliana   AGTWRVGGVLAVSRAFGDRLLKQYVVADPEIQEEKVDSSLEFLILASDGLWDVVSNEEAV 243
putative PP2C O. sativa     AGTWRVGGVLAVSRAFGDKLLKQYVVADPEIKEEIVDSSLEFLILASDGLWDVVSNKEAV 292
probable PP2C B. distachyon AGTWRVGGVLAVSRAFGDKLLKQYVVADPEIKEEVVDSSLEFLILASDGLWDVVTNDEAV 289
prot7527_c0_seq1 L. perenne AGTWRVGGVLAVSRAFGDKLLKQYVVADPEIKEEVVDGSLEFLILASDGLWDVVTNDEAV 230
                            ******************:************:** **.****************:*.***

putative PP2C A. thaliana   GMIKAIEDPEEGAKRLMMEAYQRGSADNITCVVVRFFSDQAGGIGSSSTNIPIDHGIVPD 303
putative PP2C O. sativa     DMVRPIQDPEQAAKRLLQEAYQRGSADNITVVIVRFLEGTTT-GGGPSREAASDQNS--- 348
probable PP2C B. distachyon AMVRPIEDPEQAAKGLLQEASKRGSADNITVVIVRFLEGTTEIGDGPSEEVAKDQSS--- 346
prot7527_c0_seq1 L. perenne AMVKPIEDPEQAAKRLLQEASVRGSADNITVVIVRFLDGTTTTGAGPSEEVAKDQSS--- 287
                             *:: *:***:.** *: **  ******** *:***:.  :    . * :   *:

putative PP2C A. thaliana   RISGDSST 311
putative PP2C O. sativa     -------- 348
probable PP2C B. distachyon -------- 346
prot7527_c0_seq1 L. perenne -------- 287
```

>prot5339_c0_seq1 *L. perenne*

>XP_003564321 transcription elongation factor 1 homolog *B. distachyon*

>Os07g0631100 Transcription elongation factor 1 homolog *O. sativa*

>NP_568654 transcription elongation factor 1-like protein *A. thaliana*

transcription elongation factor 1 = TEF1

```
TEF1 homolog B. distachyon  MGKRKSAAKPPPKKRMDKLDTVFSCPFCNHGSSVECRIDMKNLIGEANCRICQENFSTTV 60
TEF1 homolog O. sativa      MGKRKSAAKPPPKKRMDKLDTVFSCPFCNHGSSVECRIDMKNLIGEASCRICQENFSTTV 60
TEF1-like A. thaliana       MGKRKSRAKPAPTKRMDKLDTIFSCPFCNHGSSVECIIDMKHLIGKAACRICEESFSTTI 60
prot5339_c0_seq1 L. perenne MGKRKAAAKPPPKKRMDKLDTVFSCPFCNHGSSVECRIDMKNLIGEANCRICQESFSTTA 60
                            *****: *** *.********:************** ****.***:* ****:*.****
```

```
TEF1 homolog B. distachyon  NALTEPIDIYSEWIDECERVNTVEDDDGA-------------------------------- 89
TEF1 homolog O. sativa      NALTEPIDIYSEWIDECERVNNVEDDDGA-------------------------------- 89
TEF1-like A. thaliana       TALTEAIDIYSEWIDECERVNTAEDDVVQEEEEEVEEEEEEEEEEDDEDDHVSVKRKYNF 120
prot5339_c0_seq1 L. perenne NALTEAIDIYSEWIDECERVNTVEDDDGA-------------------------------- 89
                            .**** ***************..***
```

>prot7493_c0_seq1 *L. perenne*

>NP_974310 protein FLUORESCENT IN BLUE LIGHT *A. thaliana*

>NP_001043167 tetratricopeptide repeat(TPR)-containing protein-like Os01g0510600 *O. sativa*

>XP_003577928 PREDICTED: protein FLUORESCENT IN BLUE LIGHT, chloroplastic isoform *B. distachyon*

tetratricopeptide repeat(TPR)-containing protein-like = TPR prot-like

protein FLUORESCENT IN BLUE LIGHT = FLU

```
FLU A. thaliana             ------------------------------------------------------------ 0
TPR prot-like O. sativa     ------MELLLRPSPPPPWAI--PRRSSGERTKPCRSRSRSRTGTSKQTFPVPLLVGKVG 52
FLU B. distachyon           ------MPLLLRLRPPAAAPSPPYRRSSGCSVS---RAHLVFSETSERMFLVPSLVDQVA 51
prot7493_c0_seq1 L. perenne IFLILLMPLLMRLSPPAAAPSPPYRRSSGGYIR---RANAVLYETSEPVFP--------- 48

FLU A. thaliana             -----------------------------MWQGIGRLKL--------PVM-----AVLLT 18
TPR prot-like O. sativa     RRPFPVQCSIVRCCLSSTDAIHSTSDDIHEDNGHGHFLMKSTSDLQKVISSCFGKACLLS 112
FLU B. distachyon           RLSSCAQCSAARCVSSTMDSARPTSDGVHIANVHGHFLIKSTSDLQKAVSSCFGKAFVVS 111
prot7493_c0_seq1 L. perenne ---------LARCASSSTNHARTSSDDFDIANGHGHFLIKSTSDLQTAVSSCFGKSFVVS 99
                                                           :  *:: :         :      : :::

FLU A. thaliana             NSLQMATPLEALAAEICEPESSMFSMPILLLVALIGATVGGLLARQRKGELQRLNEQLRQ 78
TPR prot-like O. sativa     --SVMLVLPPSCFAEPCEPEYSLPNMPLLFAIAMIGATVGGLLARQRRGELKRLNDQLRQ 170
FLU B. distachyon           S-AAMLALPPSCFAESCEQGYSLPNMPLLFAIAMVGATVGGLLARQRKGELKKLNDQLRQ 170
prot7493_c0_seq1 L. perenne S-AVMLVLPPSCFAEQCEPGYSLPNMPLLFAIAMVGATVGGLLARQRKGELKKLNDQLRQ 158
                                *  .   :  ** **    *: .**:*: :*::************:***::**:****

FLU A. thaliana             INAALRRQAKIESYAPSLSYAPVGARIPDSEIIVEPKKQELISKLKTGKTFLRNQEPEKA 138
TPR prot-like O. sativa     INAALRRQAKIESYAPSLSYAPVGSKIPESEVIVDPQKDRLISYLRAGKNYLRNQAPDKA 230
FLU B. distachyon           INASLRRQAKIESYAPALSYAPAASKIPESEVIVDPQKQRLISYLRTGKNYLRNQAPDKA 230
prot7493_c0_seq1 L. perenne INASLRRQAKIESYAPALSYAPAASKVPESEVIVDPHKQRLITYLRTGKNYLRNQAPDKA 218
                            ***:************:*****..:::*:**:**:*:*:.**: *::**.:**** *:**

FLU A. thaliana             YTEFKIALELAQSLKDPTEEKKAARGLGASLQRQGKYREAIQYHSMVLAISKRESEDSGI 198
TPR prot-like O. sativa     FPEFKAAFDLAQSLGDHVEEKKAARGLGASLQRQGKYKEAIKYHSMVLNISKLTGEDAGV 290
FLU B. distachyon           FPEFKAALDLAQSLGDHVEEKKAARGLGASLQRQGNYKEAIKYHSMVLSISKMTGEDSGV 290
prot7493_c0_seq1 L. perenne FPEFKAALDLAQSLGDHVEEKKAARGLGASLQRQGNYKEAIKYHSMVLSISKMTGEDSGV 278
                            : *** *::***** * .****************:*:***:****** ***   .**:*:

FLU A. thaliana             TEAYGAIADCYTELGDLEKAGKFYDTYIARLETD     232
TPR prot-like O. sativa     TEAYGAIADCYTELGELEKAGKFYDKYIARLEND     324
FLU B. distachyon           TEAYGAIADCYTELGELEKAGKFYDEYIARLEND     324
prot7493_c0_seq1 L. perenne TEAYGAIADCYTELGELEKAGKFYDEYIARLEND     312
                            ***************:********* ******.*
```
